# Generation of Tosyl Azide in Continuous Flow Using an Azide Resin, and Telescoping with Diazo Transfer and Rhodium Acetate-Catalyzed O–H Insertion

**DOI:** 10.1021/acs.oprd.1c00377

**Published:** 2021-11-30

**Authors:** Rosella M. O’Mahony, Denis Lynch, Katie S. O’Callaghan, Stuart G. Collins, Anita R. Maguire

**Affiliations:** † School of Chemistry, Analytical and Biological Chemistry Research Facility, Synthesis and Solid State Pharmaceutical Centre, University College Cork, Cork T12 YN60, Ireland; ‡ School of Chemistry and School of Pharmacy, Analytical and Biological Chemistry Research Facility, Synthesis and Solid State Pharmaceutical Centre, University College Cork, Cork T12 YN60, Ireland

**Keywords:** diazo transfer, azide resin, sulfonyl azide generation, α-aryl-α-diazoacetate, rhodium catalysis, O−H insertion

## Abstract

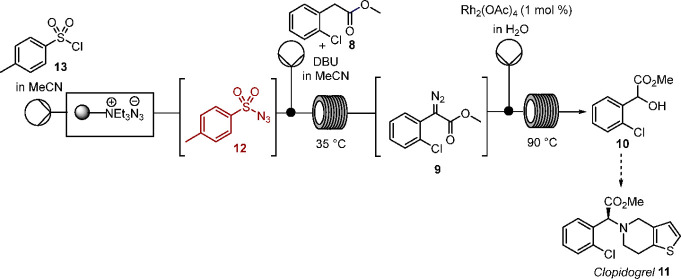

Generation of tosyl azide **12** in acetonitrile in flow under water-free conditions using an azide resin and its use in diazo transfer to a series of aryl acetates are described. Successful telescoping with a rhodium acetate-catalyzed O–H insertion has been achieved, thereby transforming the aryl acetate **8** to α-hydroxy ester **10**, a key intermediate in the synthesis of clopidogrel **11**, without requiring isolation or handling of either tosyl azide **12** or α-aryl-α-diazoacetate **9**, or indeed having significant amounts of either present at any point. Significantly, the solution of α-diazo ester **9** was sufficiently clean to progress directly to the rhodium acetate-catalyzed step without any detrimental impact on the efficiency of the O–H insertion. In addition, the rhodium acetate-catalyzed O–H insertion process is cleaner in flow than under traditional batch conditions. Use of the azide resin offers clear safety advantages and, in addition, this approach complements earlier protocols for the generation of tosyl azide **12** in flow; this protocol is especially useful with less acidic substrates.

## Introduction

While the utility and versatility of α-diazocarbonyl compounds in synthetic chemistry are clearly evident, in many instances enabling transformations which are not easily effected using other methodologies, progression of this powerful, elegant, and efficient chemistry to use at scale has been impacted by safety challenges associated with these compounds, and more particularly with their precursors (including, for example, diazoalkanes, sulfonyl azides).^1−5^ However, there have been some notable advances in this area including the generation of diazomethane at scale^6^ and N–H insertion in the Merck synthesis of thienamycin.^7^

Developments in continuous flow processing offer an excellent approach to addressing the challenges associated with use of hazardous materials.^8^ Flow chemistry has been shown to afford distinct advantages over the traditional batch approach, particularly in terms of process control; heat and mass transfer can be carried out more efficiently in tubular reactors, and the use of in-line reaction monitoring in conjunction with process automation (via feedback loops) can often be readily implemented.^9−14^ More specifically, however, the capability to generate hazardous reagents in minimal quantities in-line, immediately prior to their use, has enabled continuous platforms to improve the safety profile of the chemical processes for which they are employed.^15^

The exploitation of α-diazocarbonyl chemistry has been a key beneficiary of advances in continuous processing.^1−3,16−20^ Continuous methodologies for the generation of diazomethane have been reported,^6^ including the use of tube-in-tube reactors.^21^ Furthermore, generation and use of sulfonyl azides in continuous flow has been demonstrated, obviating the requirement to isolate and handle these potential hazardous reagents.^20,22−25^ While the Regitz diazo transfer from sulfonyl azides is a remarkably efficient and generally applicable process working with a wide variety of acceptors, the hazards associated with use and handling of sulfonyl azides are well documented.^4,5^ While modified sulfonyl azides have been developed with improved safety profiles, none of these fully resolve the concerns in effecting diazo transfer at scale using a preprepared sulfonyl azide.^4,5,26−31^ In recent years, our team has developed a number of protocols for in situ generation of sulfonyl azides followed by diazo transfer,^22^ together with telescoping with downstream reactions, including thermal Wolff rearrangement and ketene trapping,^23^ copper catalyzed C–H insertion and aromatic additions,^24^ and rhodium-mediated S–H insertion.^25^ In parallel with our work on the synthesis and use of sulfonyl azides in flow, Krasavin has described in situ generation and use of sulfonyl azides as “sulfonyl azide-free” aqueous phase diazo transfer, under traditional batch conditions.^32^

**Scheme 1:**
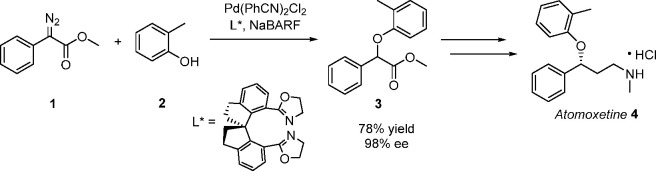
Synthesis of Atomoxetine Starting from an α-Aryl-α-diazoacetate Moiety^33^

**Scheme 2:**
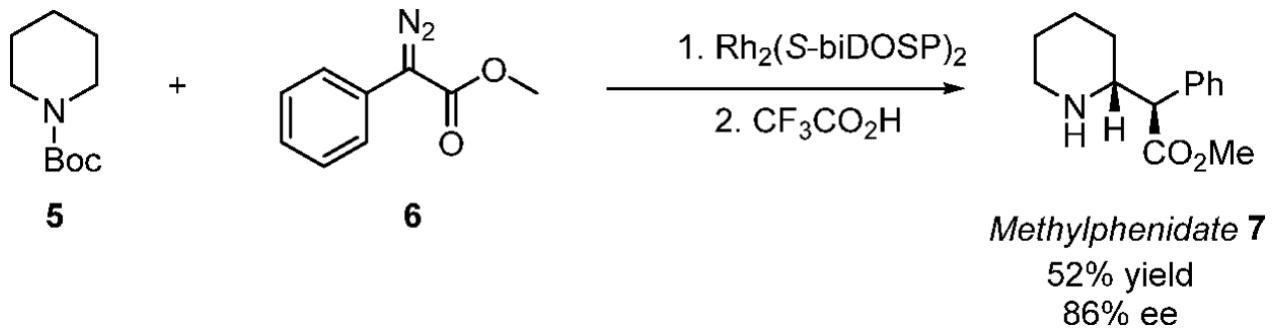
Synthesis of Methylphenidate Starting from an α-Aryl-α-diazoacetate Moiety^34^

α-Aryl-α-diazoacetates have been shown to have potential as intermediates in the synthesis of active pharmaceutical ingredients (APIs), albeit demonstrated at lab scale only to the best of our knowledge.^33,34^ Indeed, interest in the use of these compounds has prompted recent investigation and review of their thermal stability and associated hazard.^5^ For example, enantioselective palladium mediated O–H insertion of a phenyl-α-diazoacetate **1** has been employed in the synthesis of a key intermediate **3**, 98% ee, for atomoxetine **4** (Eli Lilly) in Scheme 1,^33^ while rhodium-mediated C–H insertion has led to methylphenidate **7** (Novartis) in 86% ee (Scheme 2), both of which are used to treat ADHD (Attention Deficit Hyperactivity Disorder), albeit prepared at scale by other routes.^34^

**Scheme 3:**
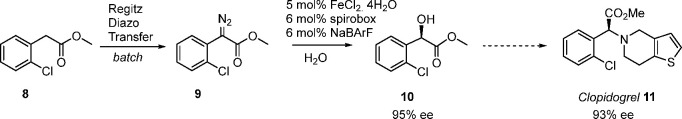
Fe-Catalyzed O–H Insertion by Zhou et al.^35^

A report from Zhou et al.^35^ employing an iron mediated enantioselective O–H insertion of an α-aryl-α-diazoacetate **9** (Scheme 3) to lead to a key intermediate **10** in a synthesis of clopidogrel **11** (a Bristol Myers Squibb API) caught our attention as an ideal application to demonstrate the synthetic potential of the in situ generation and use of sulfonyl azides for diazo transfer. While Zhou’s enantioselective O–H insertion offers an elegant route to the intermediate **10**, the challenges associated with the Regitz diazo transfer currently are likely to render this route unattractive at scale.

Accordingly, exploration of a flow process to provide the key intermediate, methyl 2-hydroxy-2-chlorophenylacetate (**10**), used in the synthesis of the antiplatelet drug clopidogrel **11** was undertaken. During the course of this work we discovered that our previously reported approach for in-line synthesis of sulfonyl azides using aqueous sodium azide was not compatible with the aryl acetate precursor, and, accordingly, a new approach was developed utilizing an azide resin as described herein, thereby expanding the sulfonyl azide in flow protocols.

## Results and Discussion

**Scheme 4:**
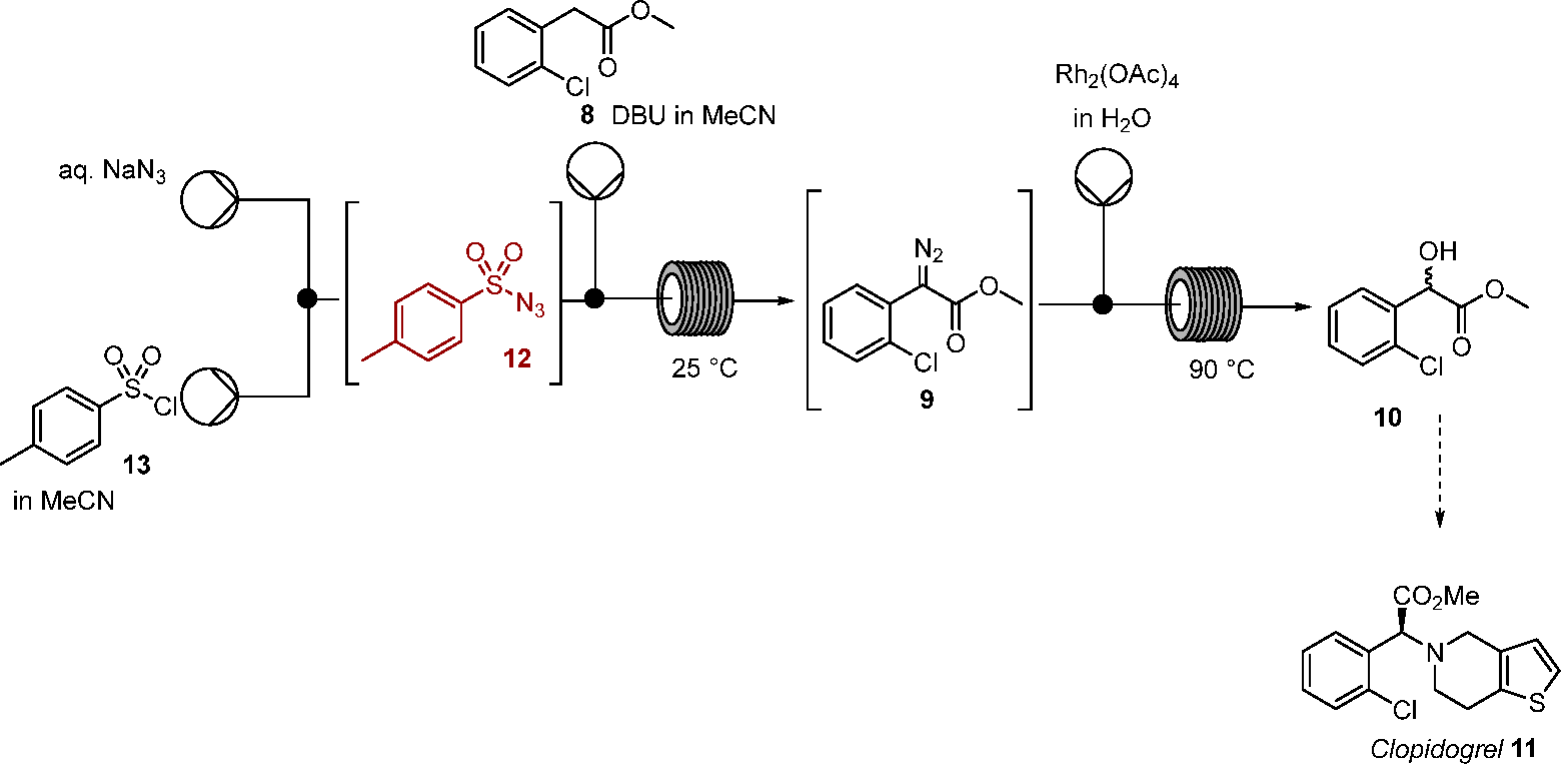
Proposed Flow Process for the Synthesis of **10** in Racemic Form

Building on our previous reports,^22−25^ formation of methyl 2-diazo-2-chlorophenylacetate (**9**) was envisaged through telescoped generation of tosyl azide **12** in aqueous acetonitrile followed by diazo transfer in continuous flow, with subsequent incorporation of rhodium-mediated O–H insertion to form **10** (Scheme 4). At the outset, the key challenge was to establish if the diazo transfer could be telescoped with a rhodium-mediated insertion into water, without isolation of the α-aryl-α-diazoacetate **9**. As our objective was to explore the feasibility of conducting the reaction sequence in flow, the use of rhodium acetate was undertaken with potential for later extension to use of enantioselective catalysts.

While telescoping sulfonyl azide synthesis and diazo transfer with a thermal Wolff rearrangement had been achieved,^23^ combination of a transition metal catalyzed process with the diazo transfer is much more challenging, in particular in terms of establishing whether the α-aryl-α-diazoacetate stream would be sufficiently clean to use directly in the rhodium-catalyzed process, or if the byproducts of the diazo transfer might poison the catalyst. In the reaction stream containing the α-aryl-α-diazoacetate there is, in addition, an equimolar amount of sulfonamide and base, presumably in part as the salt, each of which can coordinate to a rhodium catalyst and influence its reactivity.

### Diazo Transfer

Diazo transfer to the aryl ester **8** was initially investigated; as the p*K*_a_ of **8** is notably higher^36^ than the range of substrates we had previously used in our flow studies—typically involving substrates where the methylene is doubly activated by carbonyl and/or sulfonyl or sulfinyl substituents—it was important to establish at the outset that our protocol for generation and use of tosyl azide in flow was suitable for use with the aryl ester **8**, despite the reduced acidity of this substrate.

Initial attempts at diazo transfer reactions were carried out in batch by taking a solution of methyl 2-chlorophenylacetate (**8**) (0.45 M, 1 equiv) and base (1.1 equiv NEt_3_, or 1.1 equiv DBU) in acetonitrile and a solution of tosyl azide **12** (0.45 M, 1 equiv) in acetonitrile which were added simultaneously to a round-bottom flask at room temperature and stirred for 20 h, followed by concentration and analysis by ^1^H NMR spectroscopy. Using triethylamine as the base there was no evidence for diazo transfer; however, when the stronger base, DBU, was employed the reaction mixture showed a distinct and rapid color change from colorless to bright yellow in a matter of minutes, although the reaction mixture was stirred for 20 h for consistency. The ^1^H NMR spectrum of the crude product, on concentration, indicated complete diazo transfer with no unreacted ester **8** evident; following column chromatography, α-diazo aryl acetate **9** was recovered in 69% yield.

**Figure 1:**
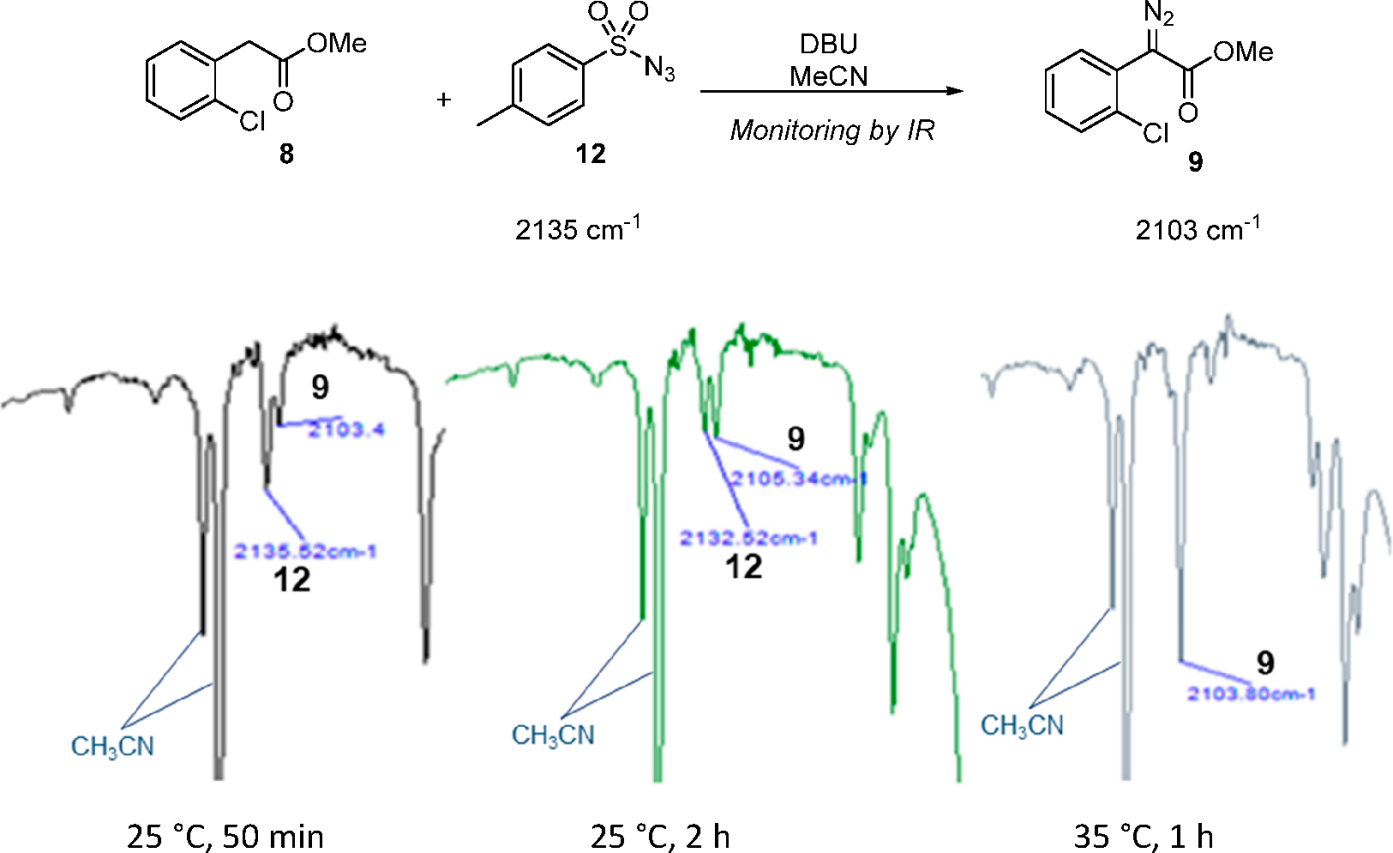
FTIR spectra of aliquots withdrawn from the diazo transfer to **8** at 25 and 35 °C. The band at ∼2104 cm^–1^ corresponds to the α-diazo ester **9**, while the band at ∼2134 cm^–1^ corresponds to tosyl azide **12**.

Wirth has described diazo transfer to aryl acetates at temperatures up to 70 °C;^17^ investigation of the impact of reaction temperature on diazo transfer to **8** was next undertaken at 25 and 35 °C. While following the reaction progress by ^1^H NMR required concentration prior to dissolution in CDCl_3_ to facilitate recording the spectrum, direct monitoring of the progress of diazo transfer was possible using FTIR (Figure 1). In practice it was evident that the rate of diazo transfer using DBU and tosyl azide in acetonitrile was substantially increased at 35 °C relative to 25 °C; the reaction proceeded more rapidly at 35 °C, and was complete within 1 h, while at 25 °C, the reaction did not reach completion within 2 h. Consequently, 35 °C was used for subsequent diazo transfer experiments in this study.

**Scheme 5:**
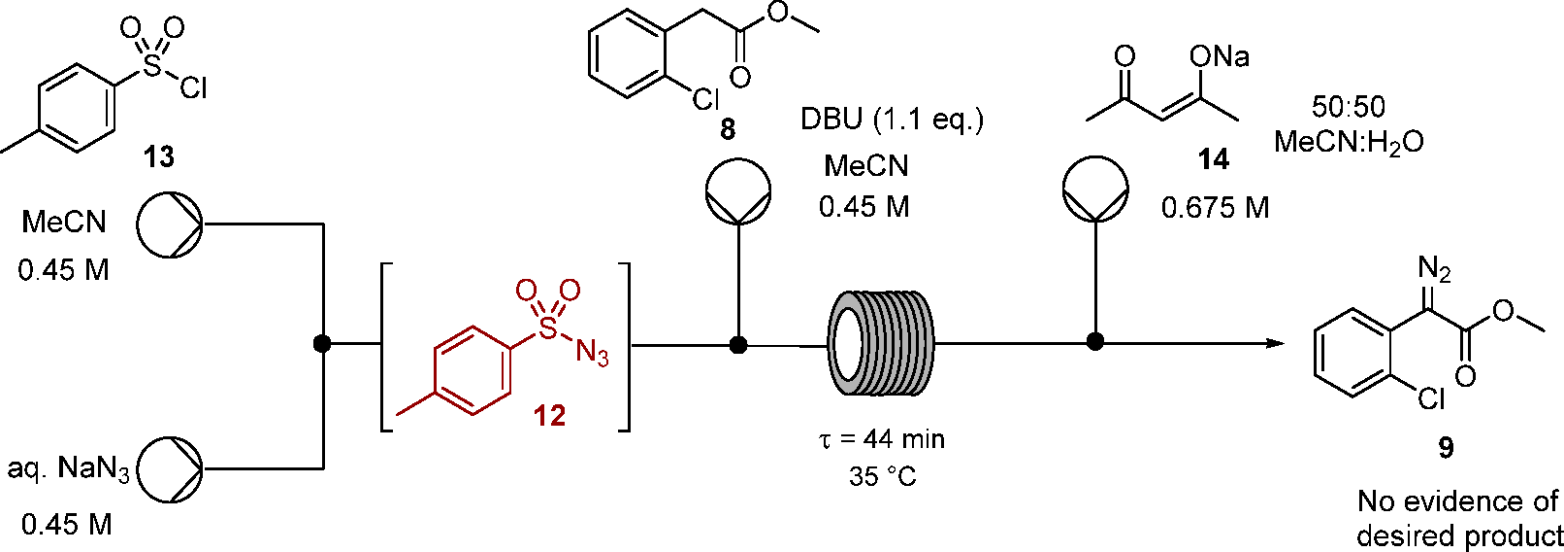
Attempted Telescoped Generation of Tosyl Azide and Diazo Transfer to **8**

Telescoping the tosyl azide **12** generation (using our protocol leading to **12** in aqueous acetonitrile) and diazo transfer in continuous flow was next attempted, utilizing DBU as the base at 35 °C (Scheme 5). Following concentration of the reaction mixture to remove acetonitrile and work up, ^1^H NMR spectroscopy confirmed that only unreacted aryl ester **8** was present, with no evidence of diazo transfer to this substrate under these conditions. Evidently, the presence of water in the reaction stream is detrimental for diazo transfer to ester **8**, which has a higher p*K*_a_ (22 for methyl phenylacetate in DMSO)^36^ than the substrates previously investigated in our earlier studies (p*K*_a_ = 10–14).^37^ The p*K*_a_ (BH^+^) of DBU in acetonitrile is reported as 24,^38^ while in water the p*K*_a_ (BH^+^) is reported as 14.^39^ It was concluded that use of aqueous sodium azide, leading to the presence of water in the reaction medium, is sufficient to reduce the basicity of DBU to the point where the diazo transfer to **8** is not efficient, although this protocol works effectively with more acidic substrates bearing two activating substituents. (Note: Tosyl azide **12** was safely quenched by treatment with sodium acetoacetonate solution as previously reported.^22^)

A diazo transfer reaction was also attempted in batch with aryl acetate **8** using NaOH (1.1 equiv) as the base instead of DBU in aqueous acetonitrile; once again there was no evidence for diazo transfer to **8** under these conditions.

While the use of aqueous sodium azide for in situ generation of sulfonyl azides had proved successful with substrates with low p*K*_a_, it is clear that for less acidic precursors an alternative approach is required. Attention next focused on developing a new protocol for generation of tosyl azide in anhydrous acetonitrile in flow to overcome this challenge. Exploration of the use of a resin loaded with azide anions was undertaken to introduce azide in the absence of water. This approach offers the additional benefit that use of an azide resin affords reduced susceptibility to handling risks, specifically associated with shock or impact.^40,41^

Two azide resins **15** and **16** were explored—one derived from Amberlite hydroxide-form ion-exchange resin **17** and one derived from Dowex chloride-form ion-exchange resin **18—**each of which was loaded by treatment with aqueous sodium azide.^41^ IR spectroscopy, conducted by placing the resin on the top plate of a UATR instrument, was used to qualitatively confirm azide loading, with the azide stretch detected at 2003 cm^–1^ for Amberlite resin **15** and 2009 cm^–1^ for Dowex resin **16**. As quantification of the azide loading was not undertaken, in subsequent use, a 3-fold excess of azide resin (assuming complete ion exchange) was employed to form tosyl azide **12** to ensure sufficient azide was present. The azide resins were prepared by stirring overnight in aqueous sodium azide solution, although use of a flow method, pumping aqueous sodium azide through a column of the resin was also undertaken.^41^

**Scheme 6:**
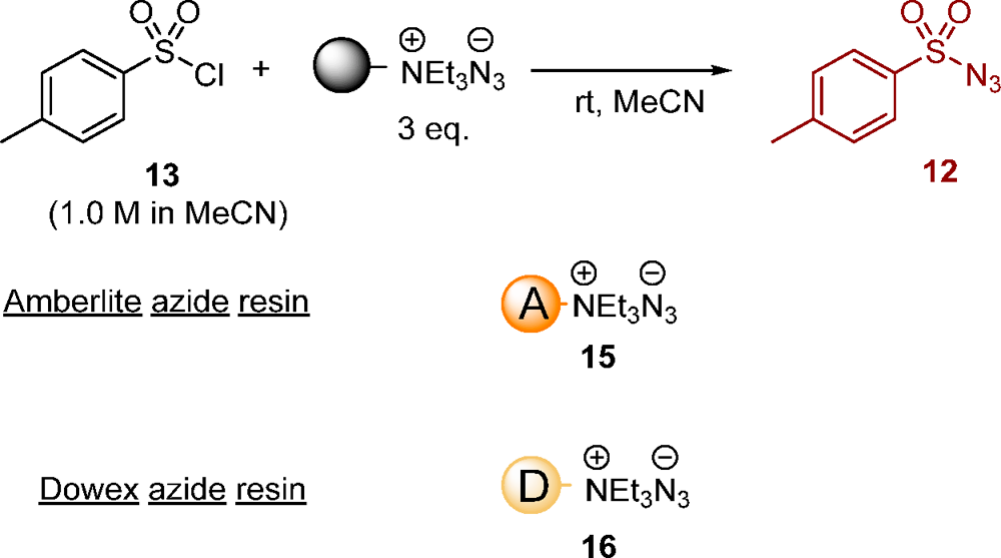
Formation of Tosyl Azide **12** in Batch Using Azide Resin **15** or **16**

To compare the two resins (Amberlite azide resin **15** or Dowex azide resin **16**), batch experiments were undertaken focusing specifically on the transformation of tosyl chloride **13** to tosyl azide **12** in acetonitrile on stirring with the azide resin, as illustrated in Scheme 6. These experiments were conducted in acetonitrile to match as closely as possible the envisaged continuous flow process, but critically in the absence of water.

**Figure 2:**
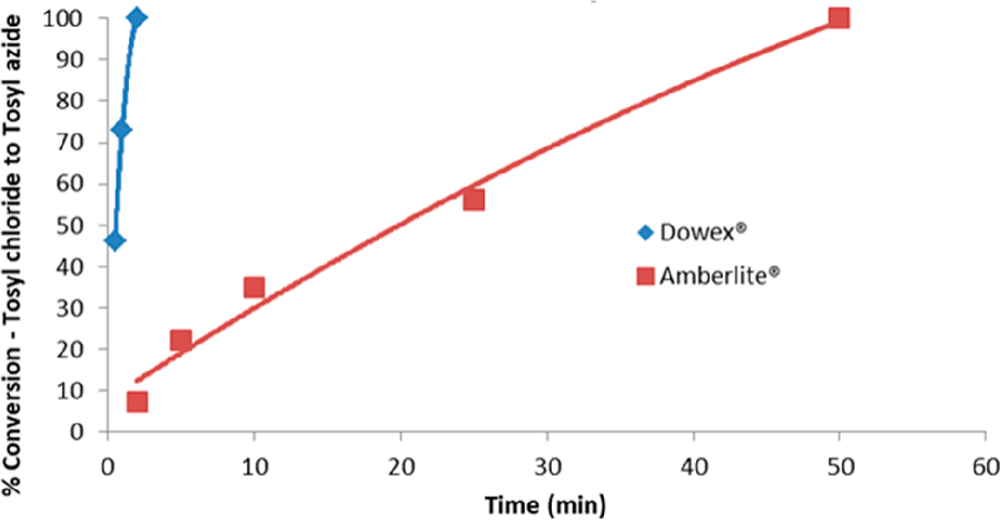
Relative rates of formation of tosyl azide **12** using either Amberlite azide resin **15** or Dowex azide resin **16** in batch, as determined by ^1^H NMR spectroscopy.

To monitor tosyl azide **12** formation, aliquots were withdrawn and the resin removed by filtration to terminate the reaction, followed by concentration and ^1^H NMR spectroscopy. In practice the rate of formation of tosyl azide **12** proved faster using the Dowex resin **16** than with the Amberlite resin **15** (Figure 2).

**Scheme 7:**
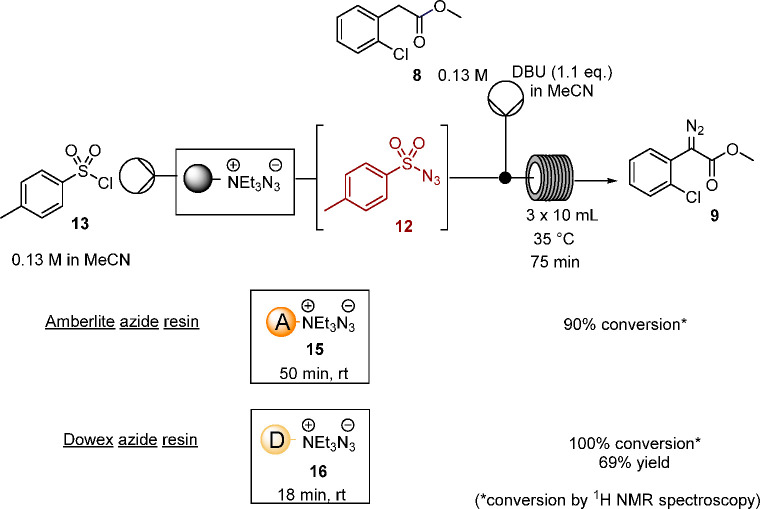
Diazo Transfer Process in Flow Using Azide Resin **15** or **16**

**Figure 3:**
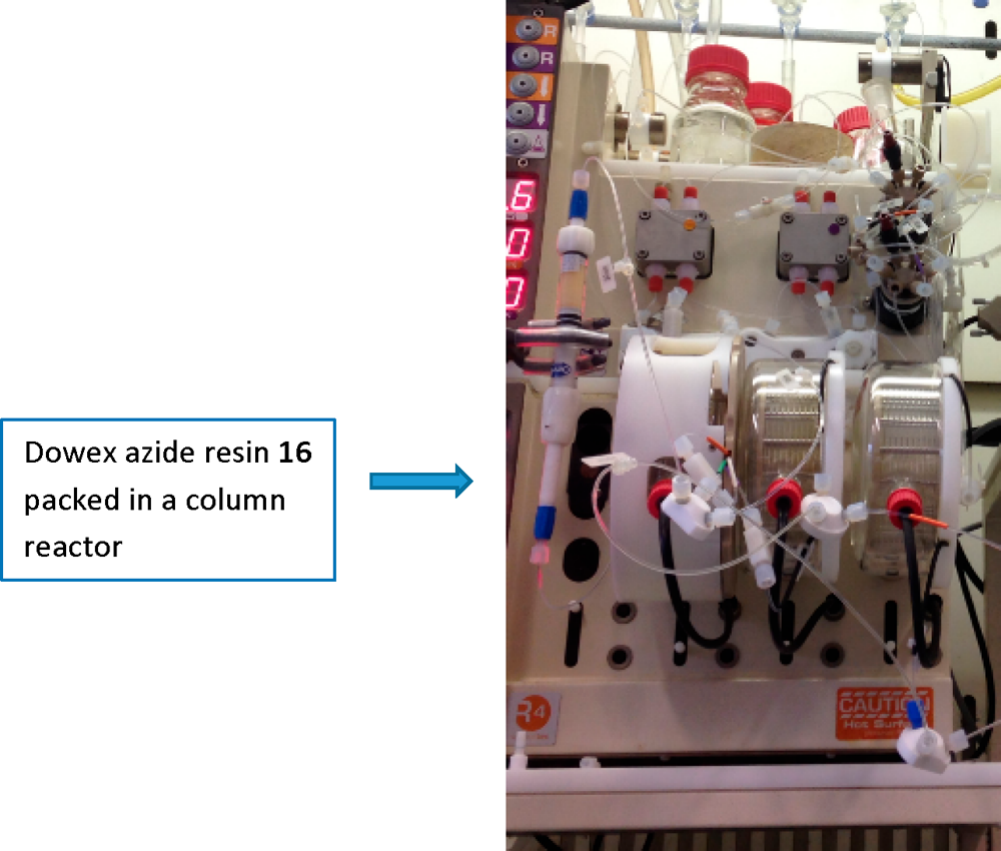
Experimental set up showing Dowex azide resin **16** packed in column.

Having established the effective formation of tosyl azide **12** on exposure to either of the azide resins, attention next focused on conducting this in flow. Thus, in situ generation of tosyl azide **12** in acetonitrile using either Amberlite azide resin **15** or Dowex azide resin **16** was performed in continuous flow (Scheme 7 and Figure 3) with a 50 or 18 min residence time, respectively allowing for the different rates of reaction that had been determined (vide supra). In this protocol the stoichiometry is readily controlled by adjusting the concentration and flow rate of tosyl chloride **13** while an excess of the azide resin is utilized. In both cases, the formation of the tosyl azide solution in acetonitrile was telescoped with diazo transfer by addition of a solution of the aryl acetate **8** (1.0 equiv), and DBU (1.1 equiv), in acetonitrile followed by passage through a reactor coil at 35 °C for 70–75 min. Concentration of the reaction outflow and ^1^H NMR spectroscopy confirmed successful diazo transfer to the aryl ester **8** in the absence of water using either resin. The use of Dowex resin **16** offered a number of advantages—in addition to the more rapid formation of tosyl azide, it was evident that complete elution of the tosyl azide **12** from this resin was more efficient than from Amberlite resin **15**, as evidenced by subsequent washings of the used resins and the differing conversion efficiencies. This protocol for the generation and use of a sulfonyl azide in a water-free reaction stream is a significant addition to our approaches for diazo transfer in flow,^19−22^ opening up diazo transfer to less acidic substrates such as aryl acetates, in addition to the advantage of introducing the azide to the process in a resin form.

As summarized in Table 1, generation of tosyl azide using Dowex resin **16** has been successfully telescoped with diazo transfer to a range of substrates including aryl acetates and diethyl malonate (**25**), adjusting the reaction conditions and specifically the reactant concentrations, relative to the preliminary investigations, to provide the α-diazo esters at higher concentration and increased throughput, while retaining a convenient residence time. In practice, this approach to the in situ generation of tosyl azide **12**, in the absence of water, proved effective for the synthesis of a series of α-diazo-α-aryl-acetates (**9**, **20**, **22**, and **24**), in addition to diethyl diazomalonate **26**. The complete diazo transfer to **25** is notable as full conversion to this product was not achieved in aqueous acetonitrile in continuous flow.^22^ Synthesis of each of the α-diazo aryl acetates (**9**, **20**, **22**, and **24**) has been previously reported diazo transfer under traditional batch conditions.^42−44^ Most importantly, this study demonstrates that diazo transfer in flow is a practical synthetic route even with substrates with relatively high p*K*_a_, such as aryl acetates; the key step which makes this approach practical is the nonaqueous medium enabled through use of the azide resin.

**Table 1:**
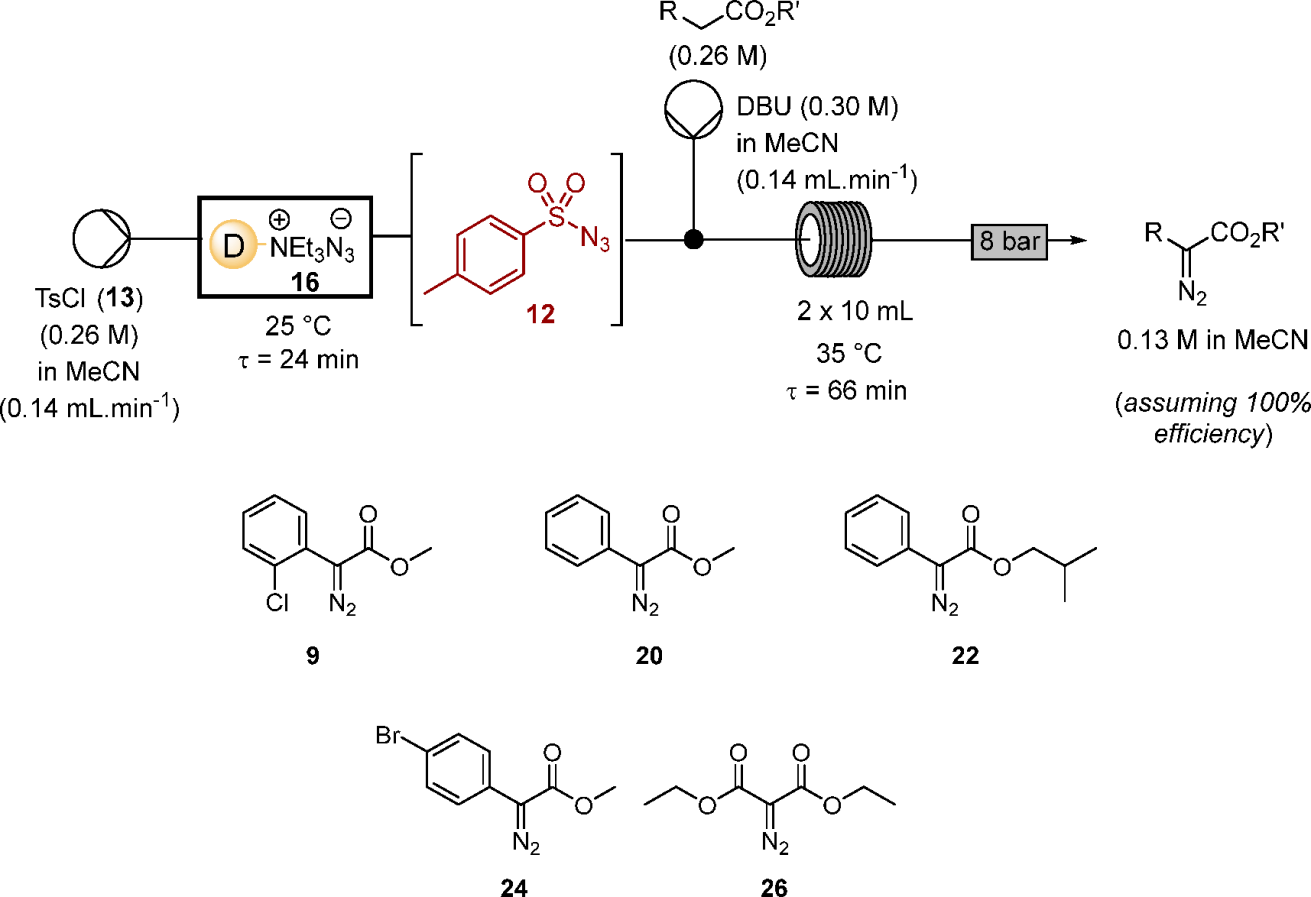
Substrate Scope of Optimized Diazo Transfer Using in Situ Generated Tosyl Azide

entry	substrate	product	R	R′	conversion (%)^a^	yield (%)^b^^,^^c^
1	**8**	**9**(42)	*o*-ClC_6_H_4_	Me	100	64 (58^d^)
2	**19**	**20**(43)	Ph	Me	100	60
3	**21**	**22**(43)	Ph	CH_2_CH(CH_3_)_2_	100	65
4	**23**	**24**(44)	*p*-BrC_6_H_4_	Me	100	58
5	**25**	**26**(48)	CO_2_Et	Et	100	62

a Conversion determined by ^1^H NMR analysis of the crude product obtained after removal of MeCN under reduced pressure

b Yield of diazo product, >90% pure by ^1^H NMR spectroscopy after chromatography.

c α-Diazo ester **9** synthesized using Dowex azide resin **16** prepared by stirring resin **18** with aqueous sodium azide (Method A, vide infra).

d α-Diazo ester **9** synthesized using Dowex azide resin **16** prepared by flowing aqueous sodium azide through the resin **18** (Method B, vide infra).^41^

Although three strategies can usually be considered to increase the productivity for a continuous process,^12,45^ a numbering-up approach,^46^ whereby multiple microreactors are run in parallel, can be envisaged as having clear advantages for this system. The use of significantly scaled-up reactors for the azide resin (stationary phase), diminishing many of the safety benefits accrued to the process through employing a continuous flow system, can readily be considered a less suitable alternative, while the evidently limited capacity of the azide resin (14.5 mL, or 8 g, of resin **16** used to generate 1 g of tosyl azide **12**) would compromise a scale-out approach,^46^ as the process cannot simply be run for longer to increase productivity. Employing parallel microreactors would also offer the potential for additional embedded process control (ideally, feedback control) to accommodate switching the reactant stream between lines (via inline valve), allowing depleted columns of resin to be replenished,^47^ while the process continues by using new/unspent columns of azide resin. For this process, integration of inline FTIR monitoring would have an obvious appeal, as depletion of azide resin should be immediately reflected in the reaction stream due to the strong characteristic sulfonyl azide stretch (2135 cm^–1^ for tosyl azide^22^) in a clear region of the spectrum.

### O–H Insertion

**Scheme 8:**
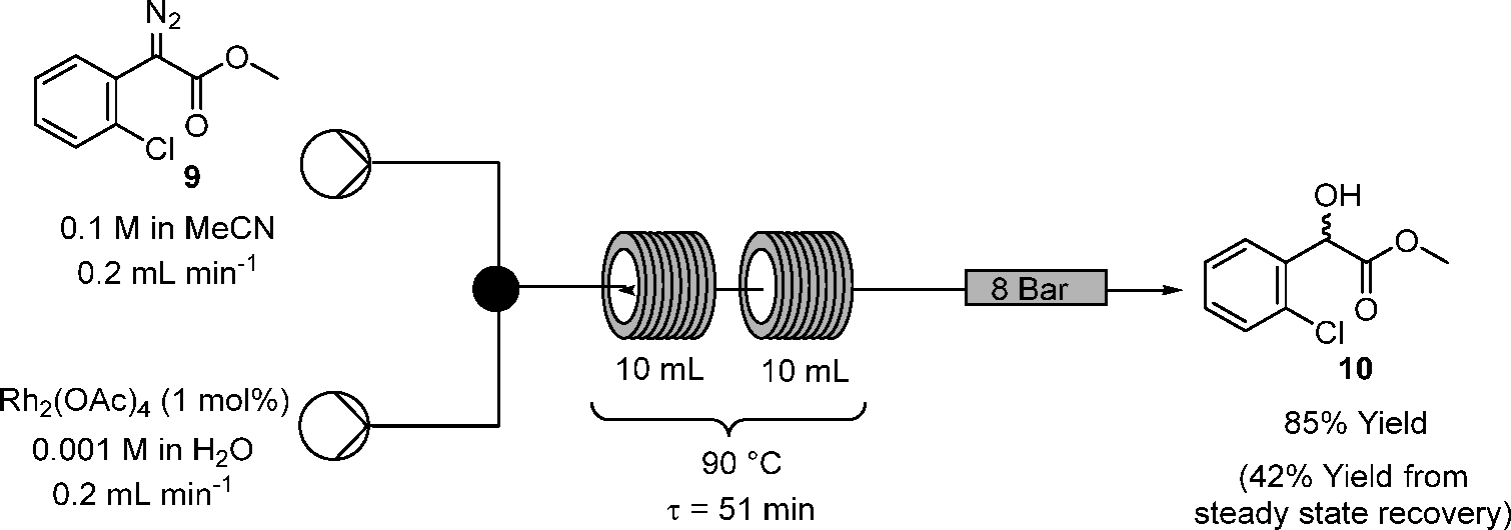
Rhodium Acetate-Catalyzed O–H Insertion Reaction of α-Diazo Aryl Acetate **9** in Flow

**Figure 4:**
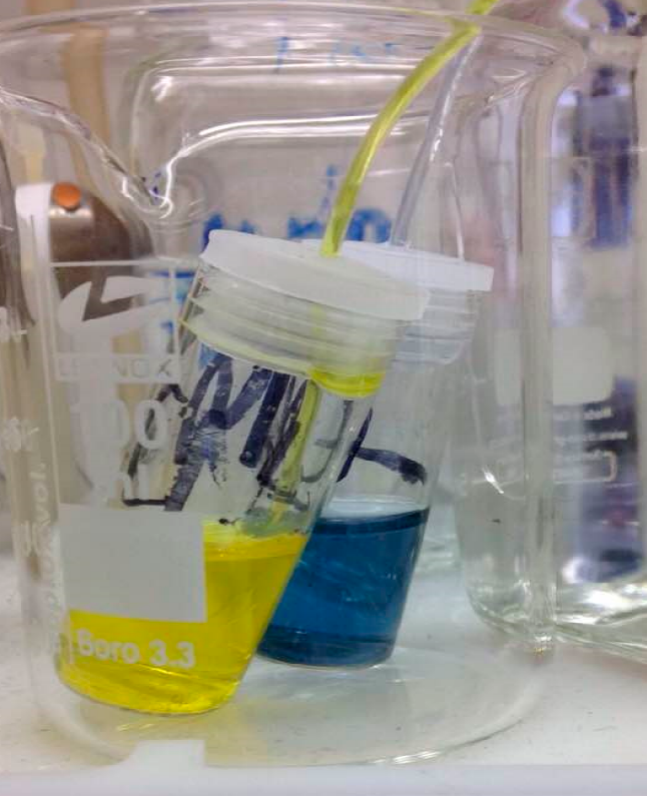
Reactant vials containing methyl 2-diazo-2-chlorophenylacetate (**9**) (yellow) and Rh_2_(OAc)_4_ (blue).

Having demonstrated that diazo transfer to form α-diazo aryl acetate **9** could be readily effected in flow, prior to attempting the telescoping with the O–H insertion step, the rhodium acetate catalyzed O–H insertion of preprepared α-aryl-α-diazoacetate **9** with water was undertaken in flow (Scheme 8 and Figure 4), utilizing an acetonitrile solution of **9** to mimic the outflow of the in situ diazo transfer process and an aqueous rhodium acetate solution.

Using the preprepared sample of α-diazo ester **9** produced in batch, a rhodium catalyzed O–H insertion was performed in flow with a residence time of 51 min at 90 °C as shown in Scheme 8. After only a few min in the reactor coil there was a color change from bright yellow to colorless, and evolution of bubbles (presumably nitrogen) was observed (see SI, Figure S2). Concentration of the reaction outflow, followed directly by ^1^H NMR spectroscopy showed that the O–H insertion product **10** was recovered in excellent quality, without requiring purification. The reaction outflow was collected only at steady state (based on the dispersion curve produced by the software used for control of the experiment—see SI for details), leading to a recovery of 42%, at this scale. When the process was repeated and all of the effluents from the reaction were collected and purified by chromatography, 85% of the hydroxy ester **10** was obtained.

**Scheme 9:**
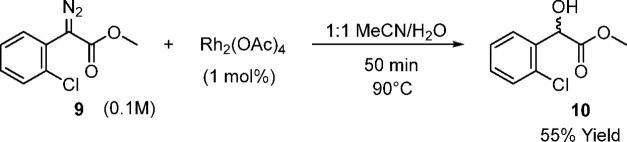
Rhodium Acetate Catalyzed O–H Insertion in Batch

To enable evaluation of the impact of conducting the reaction in flow, a batch rhodium mediated O–H insertion was undertaken under similar reaction conditions, as shown in Scheme 9. This experiment conducted by simply taking the solutions prepared at the same concentrations as for the flow experiment and physically mixing these in a round-bottom flask, which was then heated to 90 °C with stirring and then concentrated, and the crude product mixture was analyzed by ^1^H NMR spectroscopy, for comparison of quality with the concentrated outflow from the flow process.

**Figure 5:**
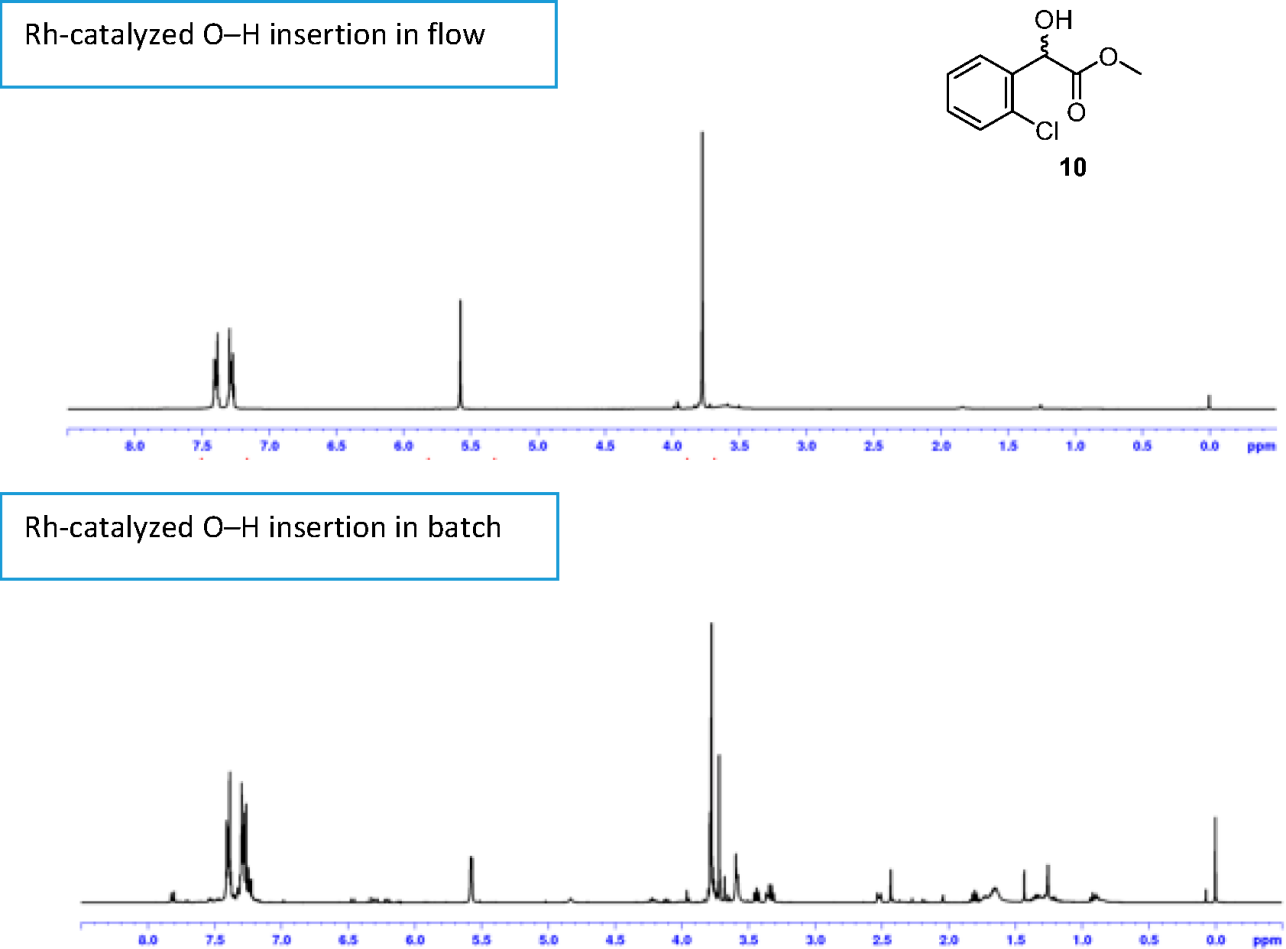
^1^H NMR (400 MHz, CDCl_3_) spectra of crude product mixtures (predominately **10**) from the rhodium-catalyzed O–H insertion to **9** in flow (Scheme 8) versus in batch (Scheme 9).

As shown in Figure 5, the ^1^H NMR spectrum of the crude product mixture from the flow process was much cleaner than that recovered from the comparable batch reaction, presumably as the α-diazo aryl acetate **9** is meeting fresh catalyst throughout the process in flow, and reaction with the α-hydroxy ester product **10** to lead to byproducts is much less likely in flow than in batch. The enhanced efficiency may also be impacted by the rapid heating to 90 °C in flow relative to the slow temperature ramp in batch.

**Scheme 10:**
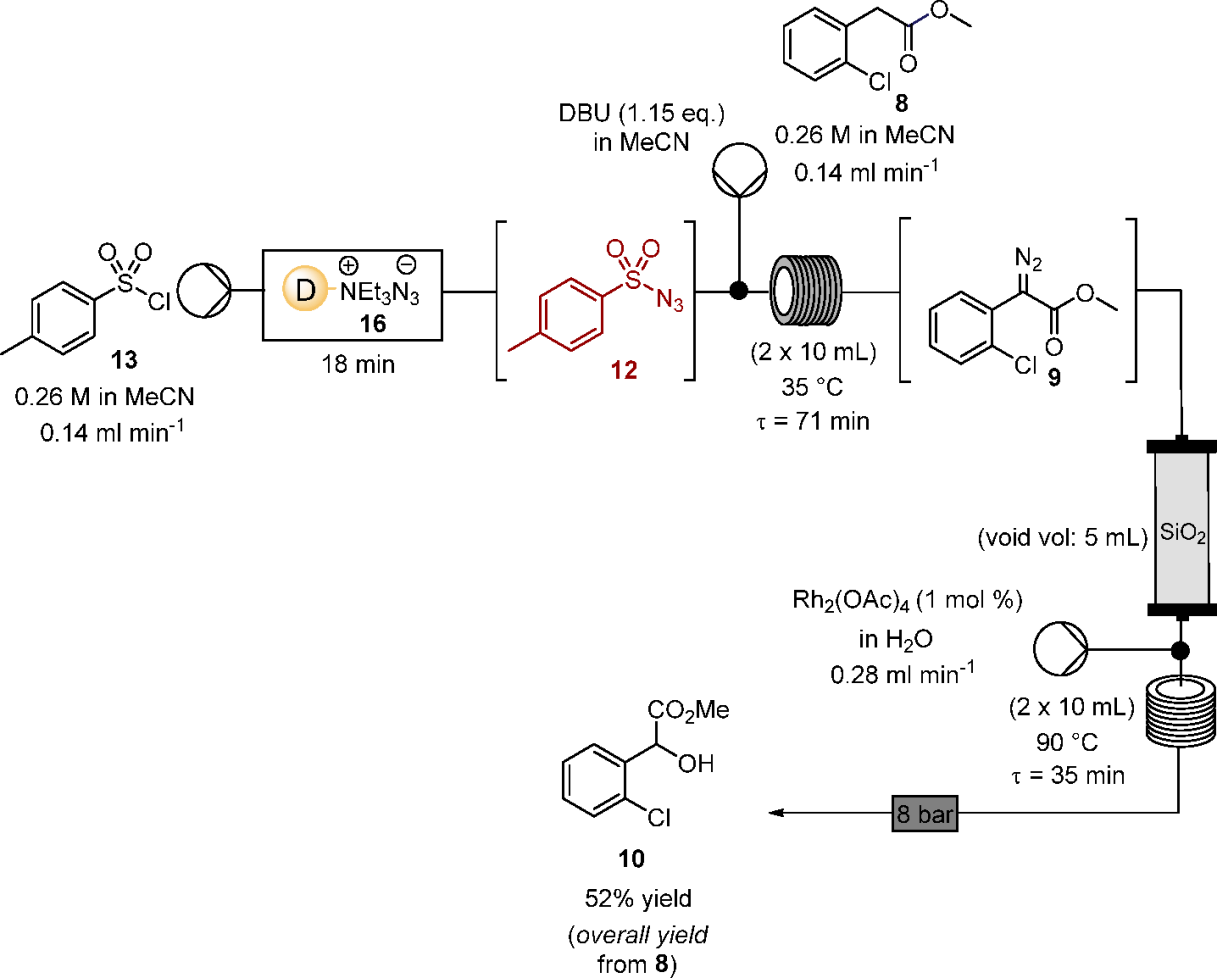
Telescoped Generation Sulfonyl Azide, Diazo Transfer to **8**, and Rhodium Acetate-Catalyzed O–H Insertion Process

Having achieved the diazo transfer and the O–H insertion in flow, the next step was to telescope the in situ formation of tosyl azide **12** and diazo transfer with the O–H insertion step. Flowing a solution of tosyl chloride **13** in water-free acetonitrile through Dowex azide resin **16** to generate a solution of tosyl azide **12** in acetonitrile proved effective, and in practice, as summarized in Scheme 7, the in situ generation of tosyl azide **12** was readily telescoped with the diazo transfer to methyl 2-chlorophenylacetate **8** leading to α-diazo aryl acetate **9**, confirming that in the absence of water, DBU is sufficiently basic to effect diazo transfer to the aryl ester in flow. On the basis of experience within the research team,^21,22^ the reaction solution containing **9**, following diazo transfer, was passed through silica gel to remove polar components including DBU and tosyl amide, possibly in part as a salt, which could impact negatively on the rhodium-catalyzed transformation. In practice, the silica gel holds some, but not all, of the toluenesulfonamide byproduct, with some elution in the polar acetonitrile medium, relative to our earlier report using dichloromethane or toluene as the solvent medium. However, this did not impact noticeably on the downstream rhodium-mediated reaction of the α-diazo aryl acetate **9**, with successful rhodium-mediated O–H insertion with water at 90 °C to form the racemic α-hydroxy ester **10**, without ever isolating or handling either tosyl azide **12** or the α-diazo aryl acetate **9**, or indeed having significant amounts of either compound present at any given time in the process (Scheme 10). The reactor effluents were collected in a round-bottom flask and then concentrated under reduced pressure which indicated the presence of the α-hydroxy ester **10** as the principal product together with some tosyl sulfonamide (see SI for ^1^H NMR spectrum of crude product mixture). Following chromatography methyl 2-(2-chlorophenyl)-2-hydroxyacetate (**10**) was isolated in 52% overall yield from **8** as a clear oil.

As the capacity of the silica gel to hold the polar components is limited, a numbering-up strategy,^46^ involving use of columns in parallel, similar to that discussed for the azide resin-mediated diazo transfer (vide supra) can readily be envisaged for increasing the productivity of the telescoped process; however, scale-up of the column size could also be considered for this component of the process.

The flow rates of the diazo product in acetonitrile and the rhodium acetate in water were maintained to ensure a 1:1 water–acetonitrile mixture on meeting at the T-piece (Scheme 10). The concentration of the α-diazo aryl acetate **9** was anticipated as 0.13 M or less (0.13 M assumes 100% efficiency), while the concentration of rhodium acetate employed was 0.001 M; accordingly the relative concentration of catalyst is comparable in the telescoped process to that in the flow process in Scheme 8, as it is reasonable to assume some minor loss of the α-diazo ester on passing through silica gel. For practical (equipment setup, see Figure S1 in the SI) reasons, the residence time in the coils for the O–H insertion reaction was limited to 35 min; interestingly, the efficiency of the O–H insertion was not negatively impacted compared to Scheme 8, where the residence time was 51 min. In addition, modest adjustments in the residence times of the azide generation and diazo transfer steps were made, also for practical reasons, when incorporating the O–H insertion into the telescoped process relative to Table 1. There may be potential to shorten the thermal exposure further, which would be beneficial if scaling up. As the efficiency of the diazo transfer under comparable conditions was 64% (Table 1, entry 1), the overall recovery of 52% for the α-hydroxy ester **10** implies that the O–H insertion process was essentially quantitative, when telescoped with the diazo transfer. This result highlights the ready combination of in situ generation of tosyl azide **12** using an azide resin and diazo transfer with downstream rhodium-mediated transformations,^22^ without any detrimental impact on the rhodium-catalyzed reaction. When conducting the telescoped experiment illustrated in Scheme 10, all of the reaction outflow was collected to provide a synthetic yield.

## Conclusion

Overall, this reaction sequence indicates that formation of tosyl azide **12** using Dowex azide resin **16**, diazo transfer, and the subsequent rhodium catalyzed transformation can be efficiently telescoped in flow to lead to the α-hydroxy ester **10**, a key intermediate in a reported synthesis of API clopidogrel **11**, without ever isolating, handling, or indeed having significant amounts of tosyl azide **12** or the α-diazo ester **9** present at any point. Notably, this has been possible with the aryl acetate substrate **8**, even though this is much less acidic than the typically doubly activated substrates we have employed to date, through use of an azide resin to generate a stream of tosyl azide **12** in acetonitrile in the absence of water. Critically, it is clear the α-diazo aryl acetate **9** can be obtained sufficiently pure to feed directly into the rhodium acetate catalyst, without catalyst poisoning—despite the presence of equimolar amounts of DBU and sulfonamide, presumably in part as a salt, at earlier stages of the reaction flow. Extension to the asymmetric synthesis of the α-hydroxy ester **10** through use of enantioselective rhodium carboxylates can be readily envisaged following this telescoped flow approach.

## Experimental Section

### General Procedures

Solvents were distilled prior to use as follows: dichloromethane was distilled from phosphorus pentoxide, ethyl acetate was distilled from potassium carbonate, hexane was stored and distilled prior to use. Diethyl ether was obtained commercially from Riedel-de-Haën and HPLC grade acetonitrile, available from Labscan Ltd., was used for diazo transfer reactions. Organic phases were dried using anhydrous magnesium sulfate. Hydroxide-form ion-exchange resin (Amberlite IRA400, 0.600–0.750 mm, ≥1.40 mequiv/mL) **17** and chloride-form ion-exchange resin (Dowex 1 × 8–200, 200–400 mesh, ≥1.2 mequiv/mL) **18** were purchased from Sigma-Aldrich. All commercial reagents were used without further purification unless otherwise stated.

^1^H (400 MHz) and ^13^C (100.6 MHz) NMR spectra were recorded on a Bruker Avance 400 MHz NMR spectrometer. ^1^H (300 MHz) and ^13^C (75.5 MHz) NMR spectra were recorded on a Bruker Avance 400 MHz NMR spectrometer. HSQC and HMBC NMR spectra were also recorded on a Bruker Avance 400 NMR spectrometer or a Bruker Avance 300 NMR spectrometer. All spectra were recorded at 300 K in deuterated chloroform (CDCl_3_) unless otherwise stated, using tetramethylsilane (TMS) as internal standard. Chemical shifts (δ_H_ and δ_C_) are reported in parts per million (ppm) relative to TMS and coupling constants (*J*) are expressed in hertz (Hz). Splitting patterns in ^1^H spectra are designated as s (singlet), d (doublet), dd (doublet of doublets), ddd (doublet of doublet of doublets), t (triplet), q (quartet) and m (multiplet). ^13^C NMR spectra were calibrated using the solvent signal, i.e., CDCl_3_: δ_C_ 77.0 ppm, and multiplicities were assigned with the aid of DEPT experiments. Assignment of ^1^H signals was aided using 2D NMR experiments including ^1^H–^1^H COSY, HSQC, and HMBC.

Infrared spectra were measured using a PerkinElmer FTIR UATR2 spectrometer.

Wet flash column chromatography was carried out using Kieselgel silica gel 60, 0.040–0.063 mm (Merck). Thin layer chromatography (TLC) was carried out on precoated silica gel plates (Merck 60 PF254). Visualization was achieved by UV (254 nm) light absorption.

All continuous processes were performed using a flow chemistry system consisting of four HPLC pumps and up to four temperature controlled tubular reactors. To prepare the reactor for operation pumps were purged with the solvent to be used in the reaction (water or acetonitrile) prior to use. All reaction tubing, coils, inlets, and connections were also purged thoroughly in a similar manner. All pumps were primed using appropriate solvents and pump backwash reservoirs were filled. The solvent that was to be used (water or acetonitrile) was flushed through all injectors and reactors. Pumps were run at reaction flow rates to check for stability, in both reagent and solvent lines, before committing reagents. Reactors were then heated to the desired temperatures to check system pressurization. General specifications of flow systems used: *Material of tubing*: PFA; *Internal diameter of tubing*: 1 mm; *Working flow rates*: 0.05 mL min^−1^ to 9.99 mL min^−1^; *Tubular reactor working volume*: 10 mL; *Temperature range*: –70 °C to 250 °C. Solid phase reagents/reaction components were employed using glass column reactors (100 mm × 10 mm internal diameter, one fixed end piece and one adjustable end piece). The number of equivalents of Amberlite azide resin **15** or Dowex azide resin **16** employed were estimated from the measured volume of these reagents used, based on the specification (mequiv/mL) of the parent resins, approximate conservation of the volume of the azide resin when generated from the parent resin, and assuming 100% azide anion exchange.

### Generation of Polymer-Bound Azides

#### Amberlite Azide Resin **15**

A solution of sodium azide (8.19 g, 126 mmol, 3 equiv) in water (100 mL) was added to a stirring mixture of Amberlite hydroxide resin **17** (30 mL, 21.46 g, 42 mmol, 1 equiv, 1.4 mmol/mL) and acetonitrile (100 mL). The reaction mixture was stirred overnight. The pH of the reaction solution was testing using indicator paper and a pH level of 10 was observed. The functionalized Amberlite azide resin **15** was washed with water (1000 mL) and acetone (500 mL). IR (UATR)/cm^–1^ 2003.

#### Dowex Azide Resin **16**

A solution of sodium azide (4.68 g, 72 mmol, 3 equiv) in water (100 mL) was added to a stirring mixture of Dowex chloride resin **18** (20 mL, 11.80 g, 24 mmol, 1 equiv, 1.2 mmol/mL) and acetonitrile (100 mL). The reaction mixture was stirred overnight. The functionalized Dowex azide resin **16** was washed with water (1000 mL) and acetone (500 mL). IR (UATR)/cm^–1^ 2009.

### Generation of Tosyl Azide Using Polymer Bound Azides

#### Batch Reaction Using Amberlite Azide Resin **15**

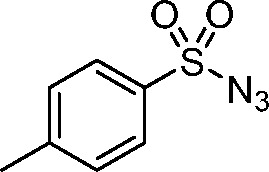



Tosyl chloride **13** (0.50 g, 2.6 mmol, 1 equiv) was added to a stirring solution of Amberlite azide resin **15** (5 mL, 3.50 g, 1.4 mequiv/mL, 7 mmol, 2.7 equiv,) in acetonitrile (25 mL) at room temperature. The reaction was sampled at 2, 5, 10, 20, and 50 min. Sampling was achieved by taking approximately 1 mL aliquots of the reaction solution at the appropriate time, followed by removal of the azide resin by filtration through glass wool. The resulting filtrate was then concentrated under reduced pressure to remove acetonitrile and a ^1^H NMR spectrum was obtained at each time point. 100% conversion was observed at 50 min. The percentage conversion was measured by integration of a tosyl chloride **13** signal [δ_H_: 7.94 (d, *J* 8.2 Hz)] vs a tosyl azide **12** signal [δ_H_: 7.85 (d, *J* 8.2 Hz)]. Spectral data was consistent with that reported in the literature.^49^

#### Batch Reaction Using Dowex Azide Resin **16**

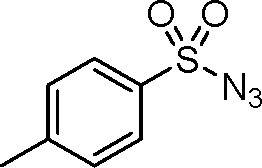



Tosyl chloride **13** (0.20 g, 1.5 mmol, 1 equiv) was added to a stirring solution of Dowex azide resin **16** (4.3 mL, 2.36 g, 1.2 mequiv/mL, 4.5 mmol, 3 equiv) in acetonitrile (15 mL) at room temperature. The reaction was sampled at 30 s, 1, 2, 5, 10, 30 min. Sampling was achieved by taking approximately 1 mL aliquots of the reaction solution at the appropriate time, followed by removal of the azide resin by filtration through glass wool. The resulting filtrate was then concentrated under reduced pressure to remove acetonitrile and a ^1^H NMR spectrum was obtained at each time point. 100% conversion was observed at 2 min. The percentage conversion was measured by integration of a tosyl chloride **13** signal [δ_H_: 7.94 (d, *J* 8.2 Hz)] vs a tosyl azide **12** signal [δ_H_: 7.85 (d, *J* 8.2 Hz)]. Spectral data was consistent with that reported in the literature.^49^

### Investigation of Diazo Transfer Reaction Conditions

#### Methyl 2-(2-chlorophenyl)-2-diazoacetate (**9**)^42^

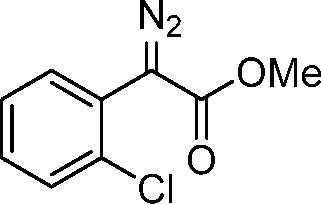



##### Attempted Preparation of **9** in Flow Using DBU in Aqueous Acetonitrile

A solution of methyl 2-chlorophenylacetate (**8**) (0.83 g, 4.5 mmol) and DBU (0.75 g, 4.95 mmol) in acetonitrile (10 mL) was prepared, and a solution of tosyl chloride **13** (0.89 g, 4.5 mmol) was made up in acetonitrile (10 mL). A solution of sodium azide (0.28 g, 4.5 mmol) was made up in water (10 mL). A solution of acetylacetone and NaOH was prepared by mixing 1.35 g of acetylacetone in approximately 9 mL acetonitrile and 0.53 g of NaOH in approximately 9 mL water and making up to the mark on a 20 mL volumetric flask with 1:1 water–acetonitrile (0.675 M) The flow system, including all HPLC pumps, was purged with respective solvents (4 mL min^–1^ for 4 min). The tosyl chloride **13** solution (9 mL, 0.45 M, 1 equiv) was pumped (0.15 mL min^–1^) into a T-piece where it met the aqueous sodium azide solution (0.15 mL min^–1^, 9 mL, 0.45 M, 1 equiv). The combined stream passed through a piece of tubing where it met the substrate **8** solution (0.15 mL min^–1^, 9 mL, 0.45 M, 1 equiv) at a T-piece. This combined stream passed through 2 × 10 mL reactor coils in series (25 °C, 44 min total residence time) before meeting a sodium acetoacetonate (**14**) solution (0.15 mL min^–1^, 9 mL, 0.675 M, 1.5 equiv) which passed through a 50 cm tube, and a back pressure regulator (8 bar). The resulting mixture was then concentrated under reduced pressure to remove acetonitrile. The crude product was extracted with diethyl ether (30 mL) and the organic layer was then washed with water (2 × 20 mL). The organic layer was dried and concentrated under reduced pressure to give the crude reaction mixture. No evidence of desired product **9** was observed by ^1^H NMR spectroscopy; only starting material **8** was recovered. The aqueous layer was safely destroyed by previously described methods.^22^

##### Preparation of **9** in Batch Using DBU in Absence of Water

A solution of methyl 2-chlorophenylacetate (**8**) (0.83 g, 4.5 mmol, 1.0 equiv) and DBU (0.75 g, 4.95 mmol, 1.1 equiv) in acetonitrile (10 mL) was added to a stirring solution of tosyl azide **12** (0.89 g, 4.5 mmol, 1 equiv) in acetonitrile (10 mL). The reaction mixture was stirred at 35 °C and was sampled by taking 1 mL aliquots and analyzed directly using IR spectroscopy. The reaction was deemed complete once the tosyl azide **12** stretch at 2135 cm^–1^ was no longer evident (1 h). The resulting mixture was then concentrated under reduced pressure to remove acetonitrile. The crude residue was purified by column chromatography, using 9:1 hexane/ethyl acetate as eluent, to afford the desired product **9** as a yellow oil (0.654 g, 69%). IR (UATR)/cm^–1^ 2096, 1703, 1479, 1434; δ_H_ (CDCl_3_, 400 MHz) 3.87 (3H, s, C*H*_3_), 7.26–7.40 (2H, m, aromatic H of phenyl group), 7.42–7.50 (1H, m, aromatic H of phenyl group), 7.53–7.59 (1H, m, aromatic H of phenyl group); δ_C_ (CDCl_3_, 100.6 MHz) 52.3 (CH_3_), 123.9 (CH), 127.2 (CH), 129.6 (CH), 130.0 (CH), 132.3 (C), 133.8 (C), 166.0 (C), no signal observed for (C=N_2_). *This reaction was also undertaken at 25 °C; slower reaction times were observed* (see Figure 2).

##### Attempted Preparation of **9** in Batch Using NaOH in Aqueous Acetonitrile

A solution of methyl 2-chlorophenylacetate (**8**) (0.73 g, 4.0 mmol, 1.0 equiv) and NaOH (0.17 g, 4.4 mmol, 1.1 equiv) in 1:1 acetonitrile–water (14 mL) was added to a stirring solution of tosyl azide **12** (0.78 g, 4.0 mmol, 1 equiv) in acetonitrile (6 mL). The reaction mixture was stirred overnight at room temperature. The resulting mixture was then concentrated under reduced pressure to remove acetonitrile. The crude product was extracted with diethyl ether (30 mL) and the organic layer was then washed with water (2 × 20 mL). The organic layer was dried and concentrated under reduced pressure to give the crude reaction mixture. No evidence of desired product **9** was observed by ^1^H NMR spectroscopy; only starting material **8** was recovered. The aqueous layer was safely destroyed by previously described methods.^22^

##### Preparation of **9** Using Amberlite Azide Resin **15** in Continuous Flow

A solution of methyl 2-chlorophenylacetate (**8**) (0.48 g, 2.6 mmol) and DBU (0.45 g, 3.0 mmol) in acetonitrile (20 mL) was prepared. A solution of tosyl chloride **13** (0.49 g, 2.6 mmol, 1 equiv) was made up in acetonitrile (20 mL). A column reactor (100 mm × 10 mm internal diameter glass column) was packed with Amberlite azide resin **15** (5.4 mL, 4.05 g, 1.4 mequiv/mL, 7.9 mmol, 3 equiv). The packed column was weighed dry. Acetonitrile was inserted into the packed column using a syringe and the column was weighed when wet with acetonitrile; the difference in mass divided by the density of acetonitrile gave the internal volume of the packed column as 3.8 mL. The flow system, including all HPLC pumps, was purged with respective solvents (4 mL min^–1^ for 4 min). The tosyl chloride **13** solution (18 mL, 0.13 M, 1 equiv) was pumped (0.07 mL min^–1^) through the polymer-bound azide **15** column (55 min residence time). The reaction stream passed through a 6 cm piece of tubing where it met the substrate **8** solution (0.07 mL min^–1^, 18 mL, 0.13 M, 1 equiv) at a T-piece. This combined stream passed into a 10 mL reactor coil (35 °C, 70 min residence time). The reaction stream passed through a 50 cm tube and back pressure regulator (8 bar). The resulting mixture was then concentrated under reduced pressure to remove acetonitrile. The ^1^H NMR spectrum of the crude product showed that the diazo transfer reaction had gone to 90% completion (9:1, diazo **9**:ester **8**). The used Amberlite azide resin **15** from the column was removed and was stirred in acetonitrile for 2 h and was filtered. The filtrate was then concentrated under reduced pressure to remove acetonitrile. Analysis by ^1^H NMR spectroscopy indicated that tosyl azide **12** was present, indicating that some of the sulfonyl azide is retained on the azide resin.

##### Preparation of **9** Using Dowex Azide Resin **16** in Continuous Flow

A solution of methyl 2-chlorophenylacetate (**8**) (0.24 g, 1.3 mmol) and DBU (0.23 g, 1.5 mmol) in acetonitrile (10 mL) was prepared. A solution of tosyl chloride **13** (0.24 g, 1.3 mmol) was made up in acetonitrile (10 mL). A column reactor (100 mm × 10 mm internal diameter glass column) was packed with Dowex azide **16** (3.8 mL, 2.36 g, 1.2 mequiv/mL, 4.5 mmol, 3.4 equiv). The packed column was weighed dry. Acetonitrile was inserted into the packed column using a syringe and the column was weighed when wet with acetonitrile; the difference in mass divided by the density of acetonitrile gave the internal volume of the packed column as 3.6 mL. The flow system, including all HPLC pumps, was purged with respective solvents (4 mL min^–1^ for 4 min). The tosyl chloride **13** solution (9 mL, 0.13 M, 1 equiv) was pumped (0.2 mL min^–1^) through the polymer-bound azide **16** column (residence time 18 min). The reaction stream passed through a 6 cm piece of tubing where it met the substrate **8** solution (0.2 mL min^–1^, 9 mL, 0.13 M, 1 equiv) at a T-piece. This combined stream passed into 3 × 10 mL reactor coils (35 °C, 75 min residence time). The reaction stream passed through a 50 cm tube and back pressure regulator (8 bar). The resulting mixture was then concentrated under reduced pressure to remove acetonitrile. The crude residue was purified by column chromatography, using 9:1 hexane/ethyl acetate as eluent, to afford the desired product **9** as a yellow oil (0.170 g, 69%) with spectral data consistent with that reported above and in the literature.^42^ The used Dowex azide resin **16** was removed from the column and stirred in acetonitrile for 2 h and was filtered. The filtrate was then concentrated under reduced pressure to remove acetonitrile. Analysis by ^1^H NMR spectroscopy indicated that no tosyl azide **12** was present.

### Diazo Transfer in Continuous Mode Using Dowex Azide Resin **16**: Substrate Scope

#### Methyl 2-(2-chlorophenyl)-2-diazoacetate (**9**)^42^

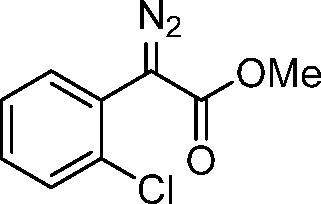



##### Method A

A solution of methyl 2-(2-chlorophenyl)acetate (**8**) (0.480 g, 2.6 mmol) and DBU (0.455 g, 3.0 mmol) in acetonitrile (10 mL) was prepared and a solution of tosyl chloride **13** (0.49 g, 2.6 mmol) was made up in acetonitrile (10 mL). A column reactor (100 mm × 10 mm internal diameter glass column) was packed with Dowex azide **16** (6 mL, 3.8 g, 1.2 mequiv/mL, 7.2 mmol, 3 equiv). The packed column was weighed dry and weighed wet with acetonitrile; the difference in mass divided by the density of acetonitrile gave the volume of the packed column as 3.6 mL. The flow system, including all HPLC pumps, was purged with respective solvents (4 mL min^–1^ for 4 min). The tosyl chloride **13** solution (9 mL, 0.26 M, 1 equiv) was pumped (0.14 mL min^–1^) through the Dowex azide **16** column (24 min residence time). The reaction stream passed through a 6 cm piece of tubing where it met the substrate **8** solution (0.14 mL min^–1^, 9 mL, 0.26 M, 1 equiv) at a T-piece. This combined stream passed into 2 × 10 mL reactor coils (35 °C, 66 min residence time). The reaction stream passed through a 50 cm tube and back pressure regulator (8 bar). The resulting mixture was then concentrated under reduced pressure to remove the solvent. The crude residue was purified by column chromatography, using 9:1 hexane/ethyl acetate as eluent, to afford the desired product **9** as a yellow oil (0.314 g, 64%) with spectral characteristics consistent with those described above and in the literature.^42^

##### Method B

A solution of methyl 2-(2-chlorophenyl)acetate (**8**) (0.480 g, 2.6 mmol) and DBU (0.455 g, 3.0 mmol, 1.15 equiv) in acetonitrile (10 mL) was prepared and a solution of tosyl chloride **13** (0.49 g, 2.6 mmol, 1 equiv) was made up in acetonitrile (10 mL). A solution of sodium azide (1.78 g) in water (25 mL) was prepared and a portion of this solution (20 mL, 1.1 M) was passed through a column reactor (100 mm × 10 mm internal diameter glass column) packed with Dowex resin **18** (5.26 mL, 3.56 g, 1.2 mequiv/mL, 6.3 mmol, 3 equiv) at 0.25 mL min^–1^. The column was then washed sequentially with water, water–MeCN (1:1) and MeCN (each pumped for 1 h at 1.0 mL min^–1^). The packed column was weighed dry and weighed wet with acetonitrile; the difference in mass divided by the density of acetonitrile gave the internal volume of the packed column as 1.32 mL. The flow system, including all HPLC pumps, was purged with respective solvents (4 mL min^–1^ for 4 min). The tosyl chloride **13** solution (8 mL, 0.26 M, 1 equiv) was pumped (0.14 mL min^–1^) through the polymer-bound azide **16** column (24 min residence time). The reaction stream passed through a 6 cm piece of tubing where it met the substrate solution (0.14 mL min^–1^, 8 mL, 0.26 M, 1 equiv) at a T-piece. This combined stream passed into 2 × 10 mL reactor coils (35 °C, 66 min residence time). The reaction stream passed through a 50 cm tube and back pressure regulator (8 bar). The resulting mixture was then concentrated under reduced pressure to remove the solvent. The crude residue was purified by column chromatography, using 9:1 hexane/ethyl acetate as eluent, to afford the desired product **9** as a yellow oil (0.251 g, 58%) with spectral characteristics consistent with those described above and in the literature.^42^

#### Methyl 2-diazo-2-phenylacetate (**20**)^43^

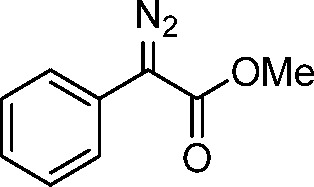



The title compound was prepared by *Method A* described for α-diazo aryl acetate **9**, above. A portion (9 mL) of a solution of methyl 2-phenylacetate (**19**) (0.390 g, 2.6 mmol) and DBU (0.455 g, 3.0 mmol) in acetonitrile (10 mL) was used to generate the diazo product **20** as a yellow oil (0.249 g, 60%). IR (UATR)/cm^–1^ 2086, 1698, 1498, 1435; δ_H_ (CDCl_3_, 400 MHz) 3.86 (3H, s, C*H*_3_), 7.14–7.21 (1H, t, *J* 7.5, aromatic H of phenyl group), 7.34–7.42 (2H, t, *J* 7.5, aromatic H of phenyl group), 7.44–7.52 (2H, d, *J* 7.5, aromatic H of phenyl group); δ_C_ (CDCl_3_, 100.6 MHz) 52.1 (CH_3_), 123.9 (CH), 125.5 (CH), 125.9 (CH), 128.9 (C), 165.6 (C), no signal observed for (C=N_2_).

#### Isobutyl 2-diazo-2-phenylacetate (**22**)^43^

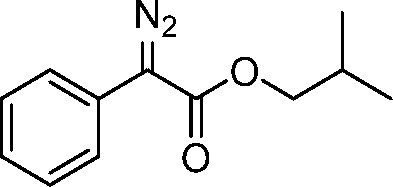



The title compound was prepared by *Method A* described for α-diazo aryl acetate **9**, above. A portion (9 mL) of a solution of isobutyl 2-phenylacetate (**21**) (0.500 g, 2.6 mmol) and DBU (0.455 g, 3.0 mmol) in acetonitrile (10 mL) was used to generate the diazo product **22** as a yellow oil (0.332 g, 65%). IR (UATR)/cm^–1^ 2080, 1700, 1498; δ_H_ (CDCl_3_, 400 MHz) 0.94–1.01 (6H, d, *J* 6.6, 2 × C*H*_3_), 1.95–2.06 (1H, m, *J* 6.6, C*H*), 4.04–4.09 (2H, d, *J* 6.6, C*H*_2_), 7.14–7.21 (1H, t, *J* 7.4, aromatic H of phenyl group), 7.35–7.42 (2H, t, *J* 7.4, aromatic H of phenyl group), 7.45–7.52 (2H, d, *J* 7.4, aromatic H of phenyl group); δ_C_ (CDCl_3_, 100.6 MHz) 19.0 (2 × CH_3_), 27.9 (CH), 70.9 (CH_2_), 123.9 (CH), 125.7 (CH), 125.8 (CH), 128.9 (C), 165.3 (C), no signal observed for (C=N_2_).

#### Methyl 2-(4-bromophenyl)-2-diazoacetate (**24**)^44^

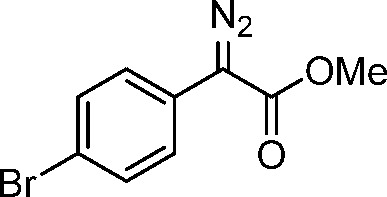



The title compound was prepared by *Method A* described for α-diazo aryl acetate **9**, above. A portion (9 mL) of a solution of methyl 2-(4-bromophenyl)acetate (**23**) (0.596 g, 2.6 mmol) and DBU (0.455 g, 3.0 mmol) in acetonitrile (10 mL) was used to generate the diazo product **24** as a yellow oil (0.351 g, 58%). IR (UATR)/cm^–1^ 3005, 2980, 2084, 1703, 1491, 490; δ_H_ (CDCl_3_, 300 MHz) 3.86 (3H, s, C*H*_3_), 7.34–7.38 (2H, d, *J* 8.9, aromatic H of phenyl group), 7.46–7.53 (2H, d, *J* 8.9, aromatic H of phenyl group); δ_C_ (CDCl_3_, 75.5 MHz) 52.1 (CH_3_), 119.4 (C), 124.7 (C), 125.3 (CH), 132.0 (CH), 165.2 (C), no signal observed for (C=N_2_).

#### Diethyl 2-diazomalonate (**26**)^48^

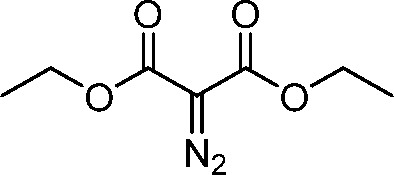



The title compound was prepared by *Method A* described for α-diazo aryl acetate **9**, above. A portion (9 mL) of a solution of diethyl malonate (**25**) (0.416 g, 2.6 mmol) and DBU (0.455 g, 3.0 mmol) in acetonitrile (10 mL) was used to generate the diazo product **26** as a yellow oil (0.271 g, 62%). ν_max_ (UATR)/cm^–1^ 2133, 1732, 1688, 1313, 1072; δ_H_ (CDCl_3_, 400 MHz) 1.32 (6H, t, *J* 7.1, 2 × CH_3_), 4.30 (4H, q, *J* 7.1, CH_2_); δ_C_ (CDCl_3_, 100.6 MHz) 14.3 (CH_3_), 61.6 (CH_2_), 161.1 (C), no signal observed for (C=N_2_).

### O–H Insertion Reaction in Flow (See Scheme 8)

#### Methyl 2-(2-chlorophenyl)-2-hydroxyacetate (**10**)^50^

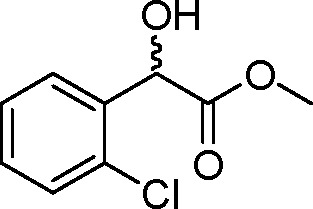



A solution of α-diazo aryl acetate **9** (0.210 g, 1.0 mmol) in acetonitrile (10 mL) was prepared and a solution of Rh_2_(OAc)_4_ (0.004 g, 0.01 mmol) was made up in water (10 mL). The flow system, including all HPLC pumps, was purged with respective solvents (4 mL min^–1^ for 4 min). The α-diazo aryl acetate **9** solution (9 mL, 0.1 M, 1 equiv) was pumped (0.2 mL min^–1^) into a T-piece where it met aqueous Rh_2_(OAc)_4_ solution (0.2 mL min^–1^, 9 mL, 0.001 M, 1 mol %). This combined stream passed into 2 × 10 mL reactor coils (90 °C, 51 min residence time). The reaction stream passed through a 50 cm tube and back pressure regulator (8 bar). The collected reaction effluents were then concentrated under reduced pressure and the crude product mixture was purified by wet flash chromatography using 9:1 hexane/EtOAc to give methyl 2-(2-chlorophenyl)-2-hydroxyacetate (**10**) as a clear oil (0.154 g, 85% yield). (UATR)/cm^–1^ 3454, 1733, 1477,1437. ^1^H NMR (CDCl_3_, 400 MHz) 3.77 (s, 3H, OC*H*_3_), 5.57 (s, 1H, C*H*OH), 7.25–7.31 (2H, m, aromatic H), 7.37–7.43 (2H, m, aromatic H). ^13^C NMR (CDCl_3_, 100.6 MHz) 53.2 (CH_3_), 70.4 (CH), 127.2 (CH), 128.9 (CH), 129.8 (CH), 130.0 (CH), 133.5 (C), 136.0 (C), 173.7 (C). *When the process was separately undertaken with only the steady state of the reaction stream collected* (based on the dispersion curve on the software used for control of the experiment—see SI for details), *the resulting mixture was then concentrated under reduced pressure to remove acetonitrile and α-hydroxy ester***10***was recovered cleanly* (see Figure 5) *as a clear oil (0.077 g, 42% yield)*.

### O–H Insertion Reaction in Batch (See Scheme 9)

#### Methyl 2-(2-chlorophenyl)-2-hydroxyacetate (**10**)^50^

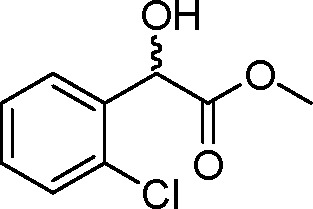



A solution of α-diazo aryl acetate **9** (0.210 g, 1.0 mmol, 1 equiv) in acetonitrile (10 mL) was prepared and was added to a stirring aqueous solution of Rh_2_(OAc)_4_ (0.004 g in 10 mL, 1 mol %) at room temperature. The reaction mixture was heated to 90 °C and stirred for 51 min for direct comparison to the corresponding flow reaction. The resulting mixture was then concentrated under reduced pressure and the crude product mixture The crude product was purified by wet flash chromatography using 9:1 hexane/EtOAc to give methyl 2-(2-chlorophenyl)-2-hydroxyacetate (**10**) as a clear oil (0.110 g, 55% yield).

### Telescoped Generation of Sulfonyl Azide, Diazo Transfer, and Rhodium Acetate-Catalyzed O–H Insertion Reaction in Flow (See Scheme 10)

#### Methyl 2-(2-chlorophenyl)-2-hydroxyacetate (**10**)^50^

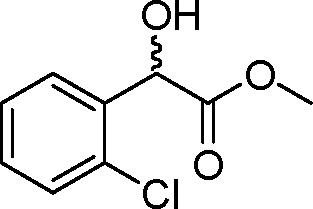



A solution of methyl 2-chlorophenylacetate (**8**) (0.480 g, 2.6 mmol) and DBU (0.455 g, 3.0 mmol, 1.15 equiv) in acetonitrile (10 mL) was prepared and a solution of tosyl chloride **13** (0.490 g, 2.6 mmol) was made up in acetonitrile (10 mL). A solution of Rh_2_(OAc)_4_ (0.006 g, 1 mol %) was made up in water (10 mL). A column reactor (100 mm × 10 mm internal diameter glass column) was packed with Dowex azide **16** (6 mL, 3.8 g, 1.2 mequiv/mL, 7.2 mmol, 3.0 equiv). The packed column was weighed dry and weighed wet with acetonitrile; the difference in mass divided by the density of acetonitrile gave the internal volume of the packed column as 2.86 mL. A packed column of silica (2.45 g) was also prepared. The column was weighed dry and weighed wet with acetonitrile; the difference in mass divided by the density of acetonitrile gave the internal volume of the packed column as 5.0 mL. The flow system, including all HPLC pumps, was purged with the appropriate solvents (4 mL.min^–1^ for 4 min). The tosyl chloride **13** solution (9 mL, 0.26 M, 1 equiv) was pumped (0.14 mL min^–1^) through the Dowex azide **16** column (7.2 mmol, 2.86 mL internal volume, 18 min residence time). The reaction stream passed through a 6 cm piece of tubing where it met the substrate **8** solution (0.14 mL min^–1^, 9 mL, 0.26 M, 1 equiv) at a T-piece and passed through 2 × 10 mL reactor coils (35 °C, 71 min residence time). The stream containing α-diazo aryl acetate **9** then passed through the silica gel column for removal of DBU (18 min). The reaction stream passed through a 6 cm piece of tubing where it met the rhodium acetate solution (0.28 mL min^–1^, 8 mL) at a T-piece and passed through 2 × 10 mL reactor coils (90 °C, 35 min). The reaction stream passed through a 50 cm tube and back pressure regulator (8 bar). The reactor effluents were all collected in a round-bottom flask and then concentrated under reduced pressure. The crude product was purified by wet flash chromatography using 7:3 hexane/EtOAc to give the α-hydroxy ester **10** as a clear oil (0.226 g, 52% yield) with spectral data consistent with that reported above and in the literature.^50^*Note: Yield is based on recovery of pure***10***(following chromatography) from initial substrate***8**, *incorporating the tosyl azide generation diazo transfer, removal of DBU and O–H insertion*.

## Supporting Information Available

Details of continuous flow platforms and setup, supplementary figures, copies of ^1^H and ^13^C NMR spectra (PDF)
