# Supplementary material for: Generation of Tosyl Azide in Continuous Flow Using an Azide Resin, and Telescoping with Diazo Transfer and Rhodium Acetate-Catalyzed O–H Insertion
Source: Org Process Res Dev. 2021 Nov 30;25(12):2772–85. doi: 10.1021/acs.oprd.1c00377 (PMC8689650; doi:10.1021/acs.oprd.1c00377)
Supplement: Supplementary file 1 — op1c00377_si_001.pdf [file op1c00377_si_001.pdf]

Supporting Information

Generation of Tosyl Azide in Continuous Flow using an Azide Resin, and Telescoping with Diazo Transfer and Rhodium Acetate-Catalyzed O–H Insertion

Rosella M. O'Mahony[†], *Denis Lynch*[†], *Katie S. O'Callaghan*[†], *Stuart G. Collins*[†*], *and Anita R. Maguire*[‡ *]

[†] *School of Chemistry, Analytical and Biological Chemistry Research Facility, Synthesis and Solid State Pharmaceutical Centre, University College Cork, Cork T12 YN60, Ireland*
[‡] *School of Chemistry and School of Pharmacy, Analytical and Biological Chemistry Research Facility, Synthesis and Solid State Pharmaceutical Centre, University College Cork, Cork T12 YN60, Ireland*
**Email: a.maguire@ucc.ie; *Email: stuart.collins@ucc.ie*

Table of Contents

Details of Continuous Flow Platforms & Set up S2

Supplementary Figures S3

Copies of ^{1}H and ^{13}C NMR Spectra S4

References S12

Details of Continuous Flow Platforms & Set up

Continuous processes were performed using a Vapourtec R-Series flow system consisting of four piston (HPLC) pumps. Solid phase reagents/reaction components were employed using Omnifit glass column reactors (100 mm × 10 mm internal diameter, one fixed end piece and one adjustable end piece). For processes where the product stream was collected only while at steady-state, this was determined by the proprietary software (Flow Commander) installed on the flow chemistry system.

Table S1. General specifications for Vapourtec R-Series system

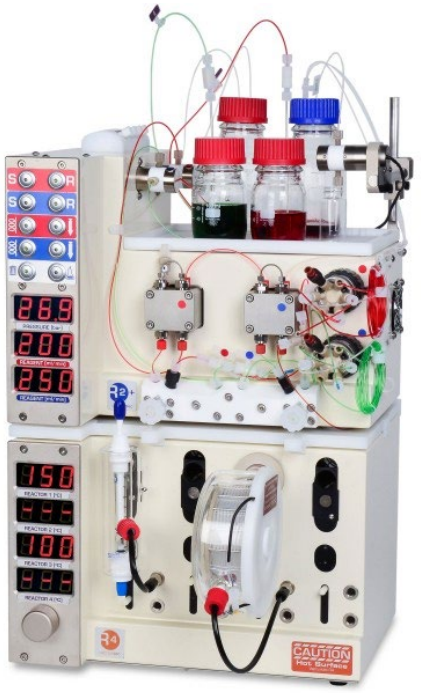

General specifications for continuous-flow system	
Material of tubing	PFA
Internal diameter of tubing	1 mm
External diameter of tubing	1.59 mm
Working flow rates	0.05 mL/min – 9.99 mL/min
Tubular reactor working volume	10 mL
Temperature range	–70 ºC to 250 ºC

Supplementary Figures

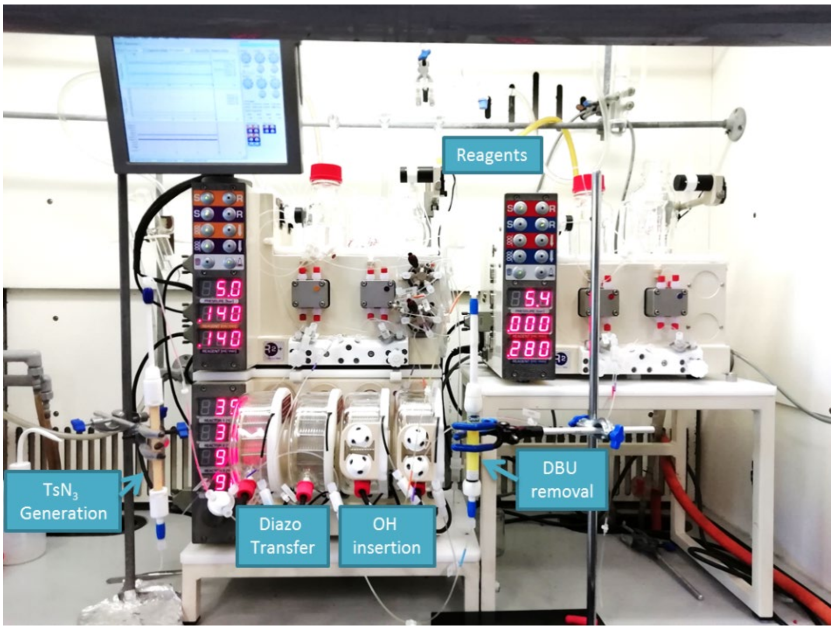

Figure S1. System configuration for telescoped generation of tosyl azide, diazo transfer and O–H insertion (see Scheme 10).

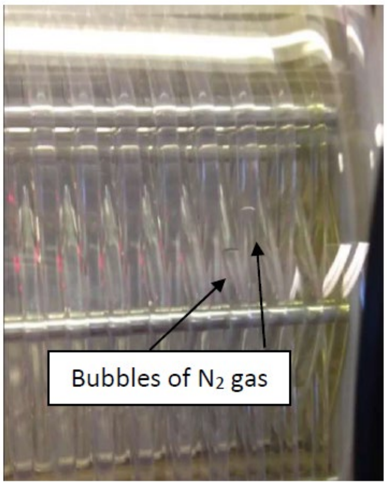

Figure S2. Nitrogen bubbles released in reactor coil during rhodium acetate-catalyzed O–H insertion reaction of α-diazo aryl acetate **9** in flow (see Scheme 8).

Copies of ^1H and ^{13}C NMR Spectra

NMR spectra of the following compounds were in agreement with those previously reported:

Methyl 2-(2-chlorophenyl)-2-diazoacetate (9)[1]

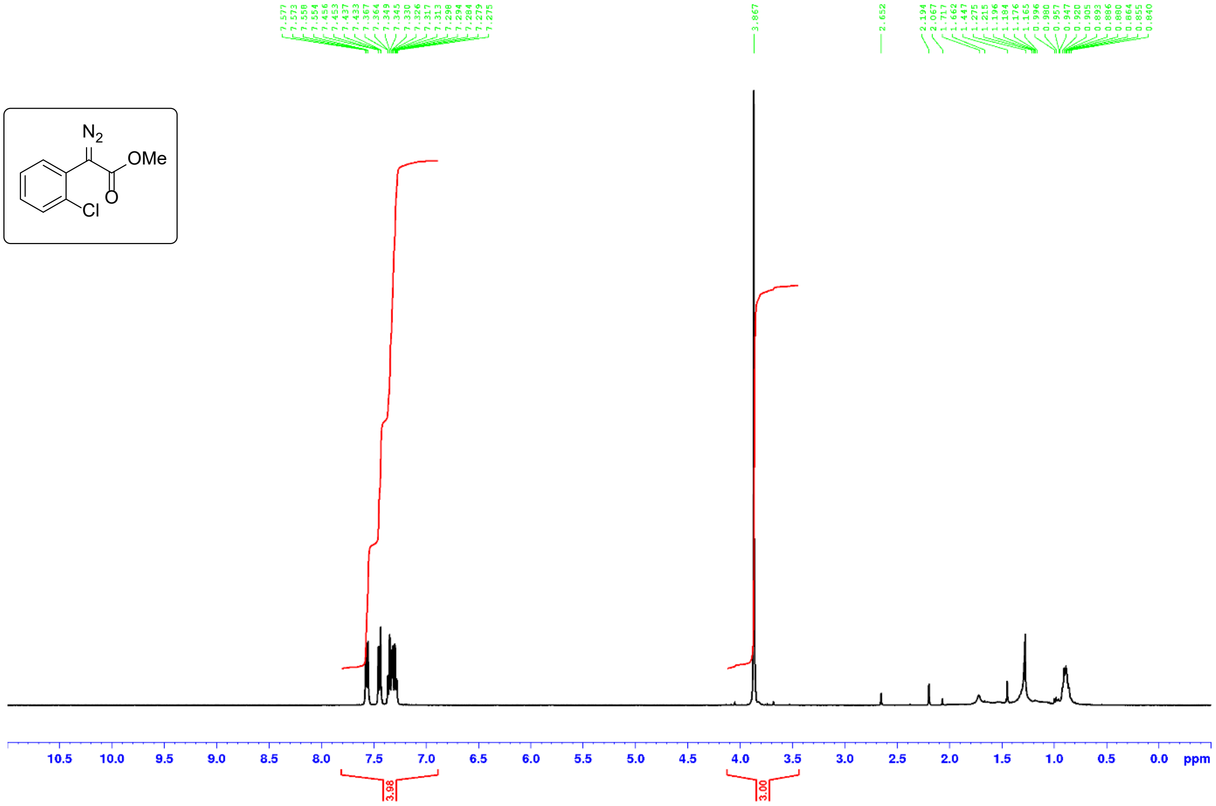

Figure S3. ^{1}H NMR ($CDCl_3$, 400 MHz) spectrum of α-diazo ester **9**.

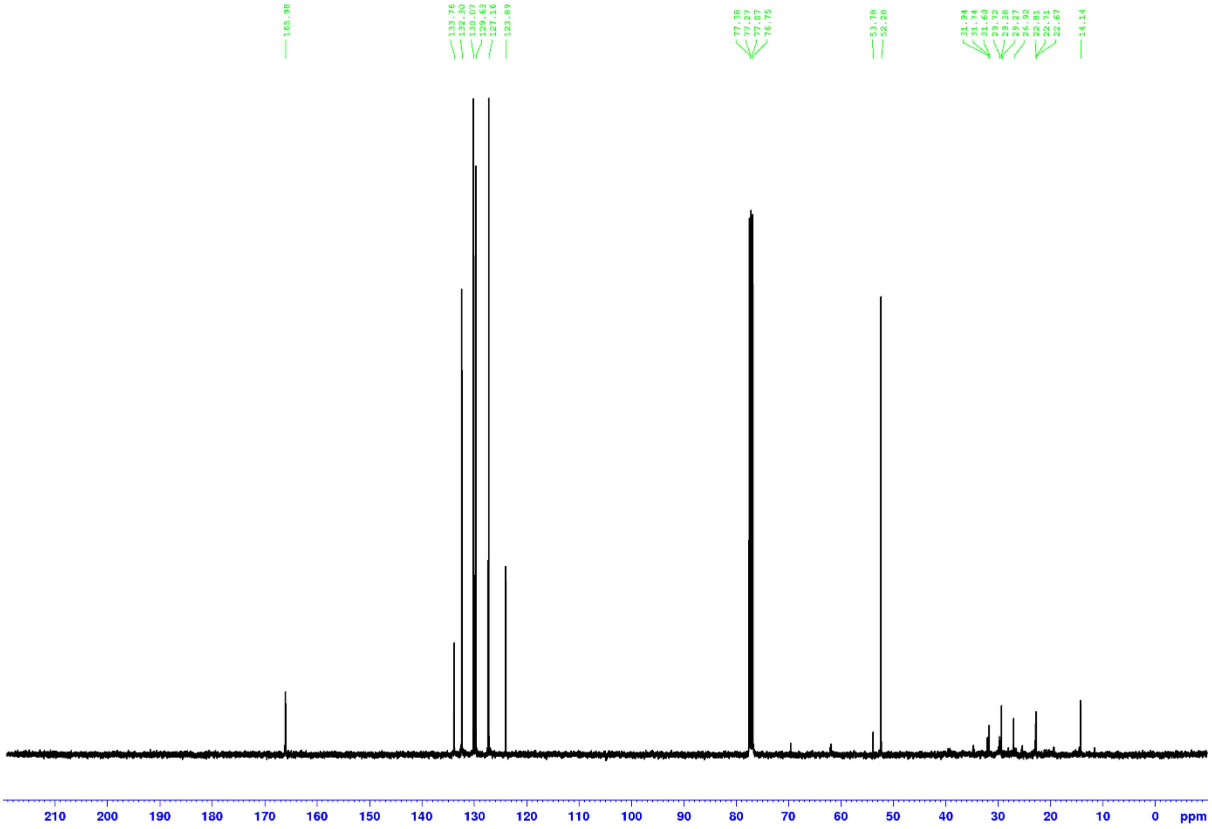

Figure S4. $^{13}C\{^{1}H\}$ NMR ($CDCl_3$, 100.6 MHz) spectrum of α-diazo ester **9**.

Methyl 2-diazo-2-phenylacetate (20)[2]

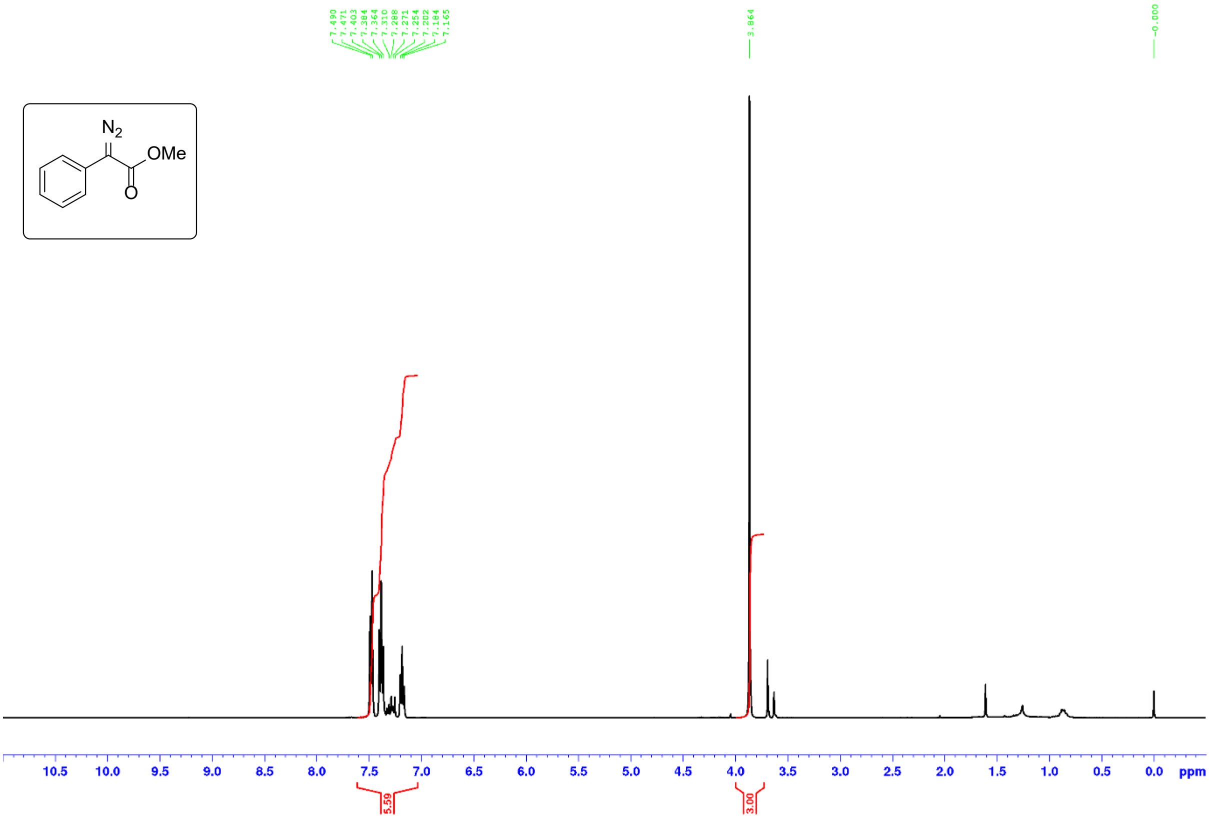

Figure S5. ^{1}H NMR ($CDCl_3$, 400 MHz) spectrum of α-diazo ester **20**.

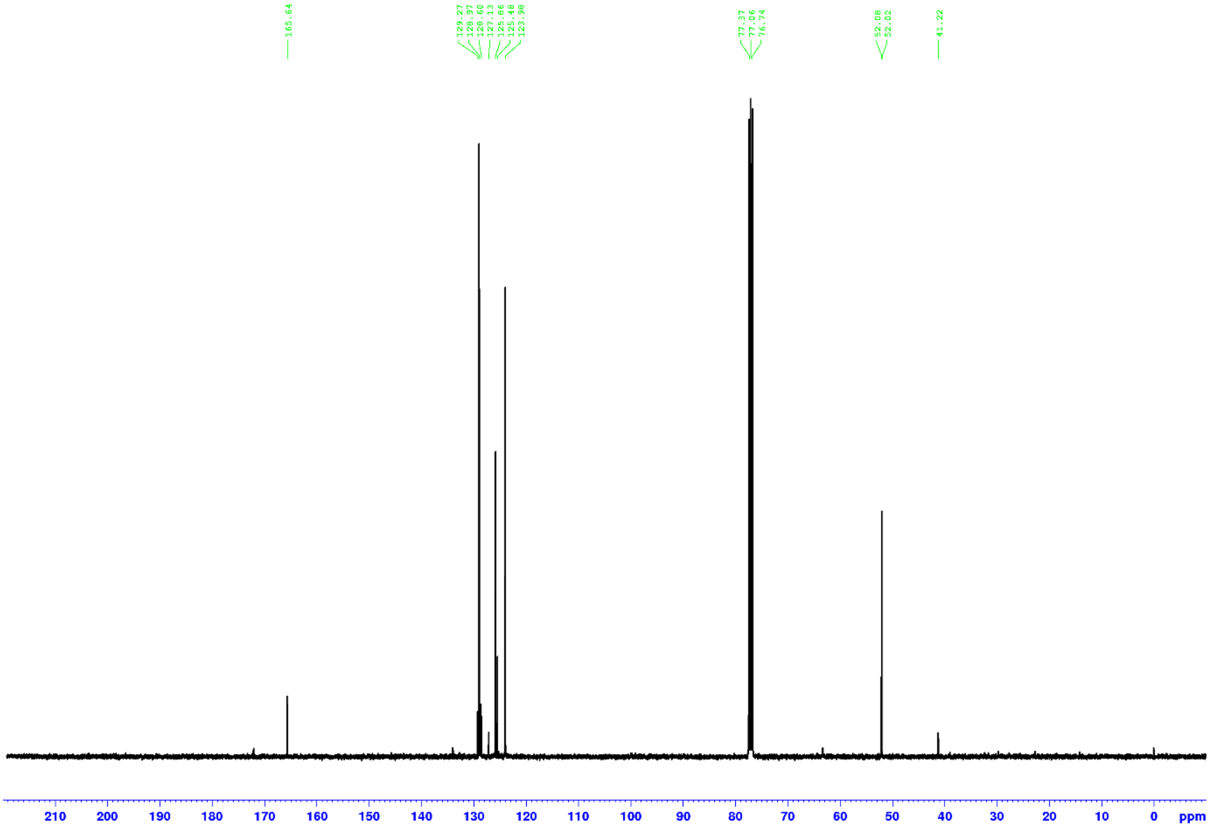

Figure S6. ^{13}C{^{1}H} NMR ($CDCl_3$,100.6 MHz) spectrum of α-diazo ester **20**.

Isobutyl 2-diazo-2-phenylacetate (22)[2]

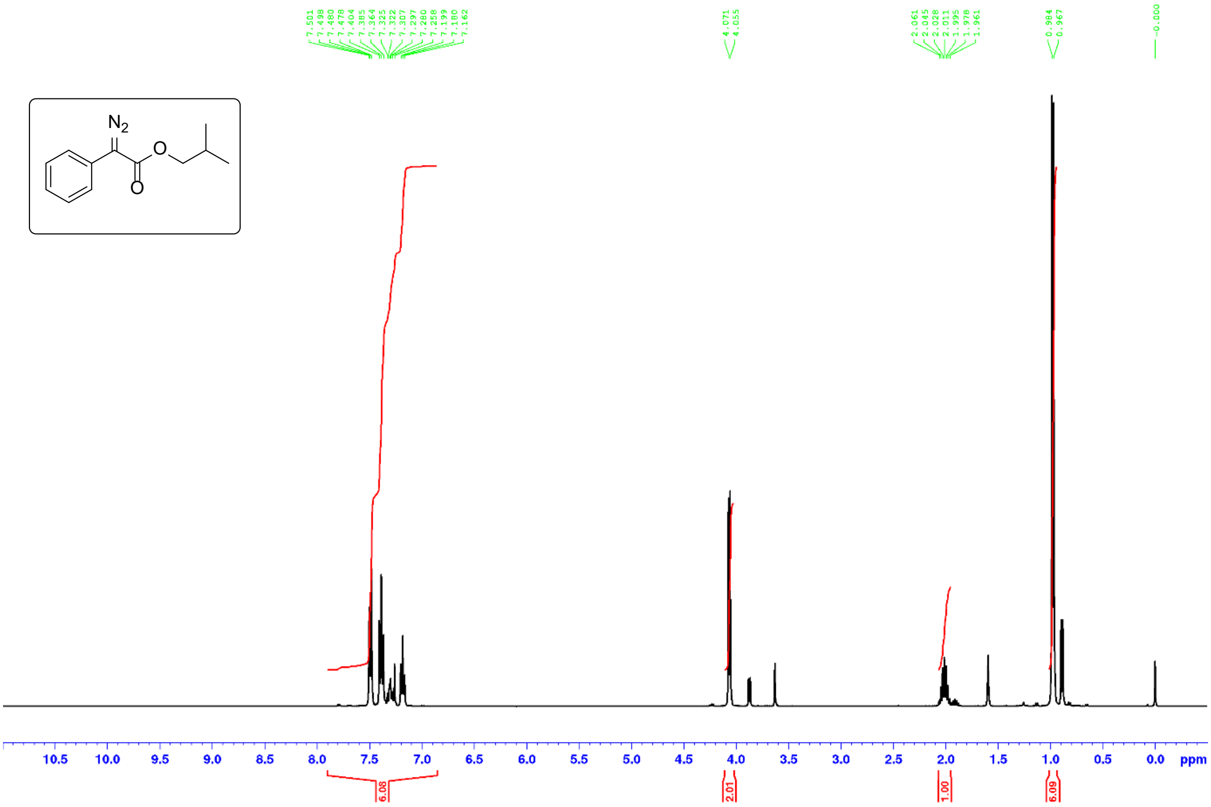

Figure S7. ^{1}H NMR ($CDCl_3$, 400 MHz) spectrum of α-diazo ester **22**.

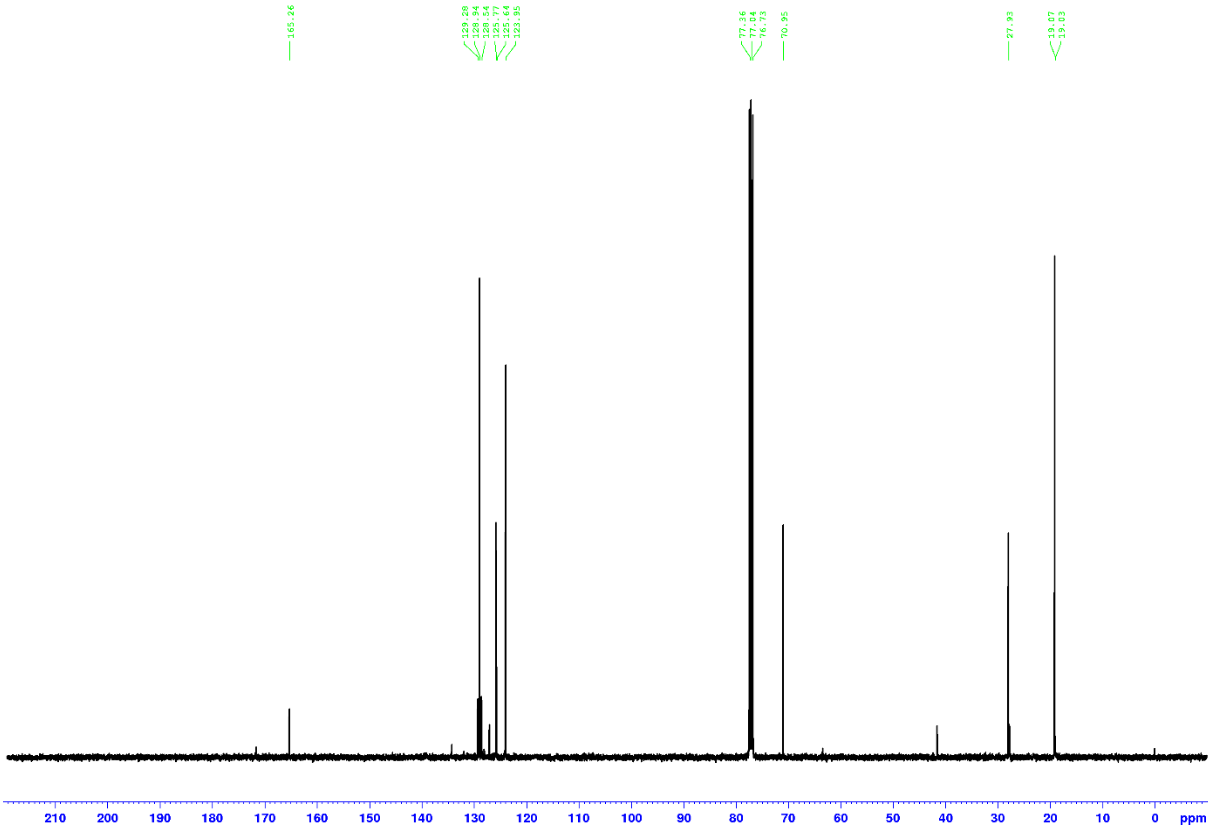

Figure S8. $^{13}C\{^{1}H\}$ NMR ($CDCl_3$, 100.6 MHz) spectrum of α-diazo ester **22**.

Methyl 2-(4-bromophenyl)-2-diazoacetate (24)[3]

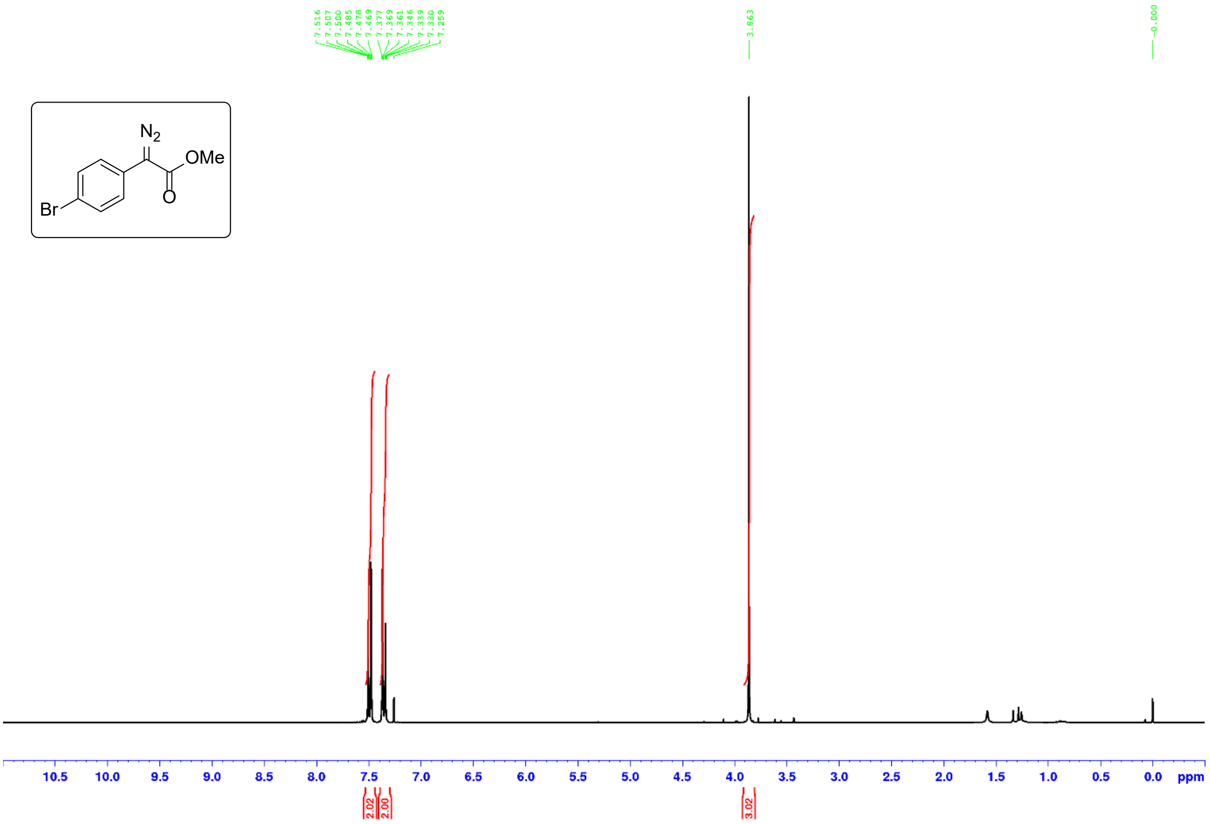

Figure S9. ^{1}H NMR ($CDCl_3$, 300 MHz) spectrum of α-diazo ester **24**.

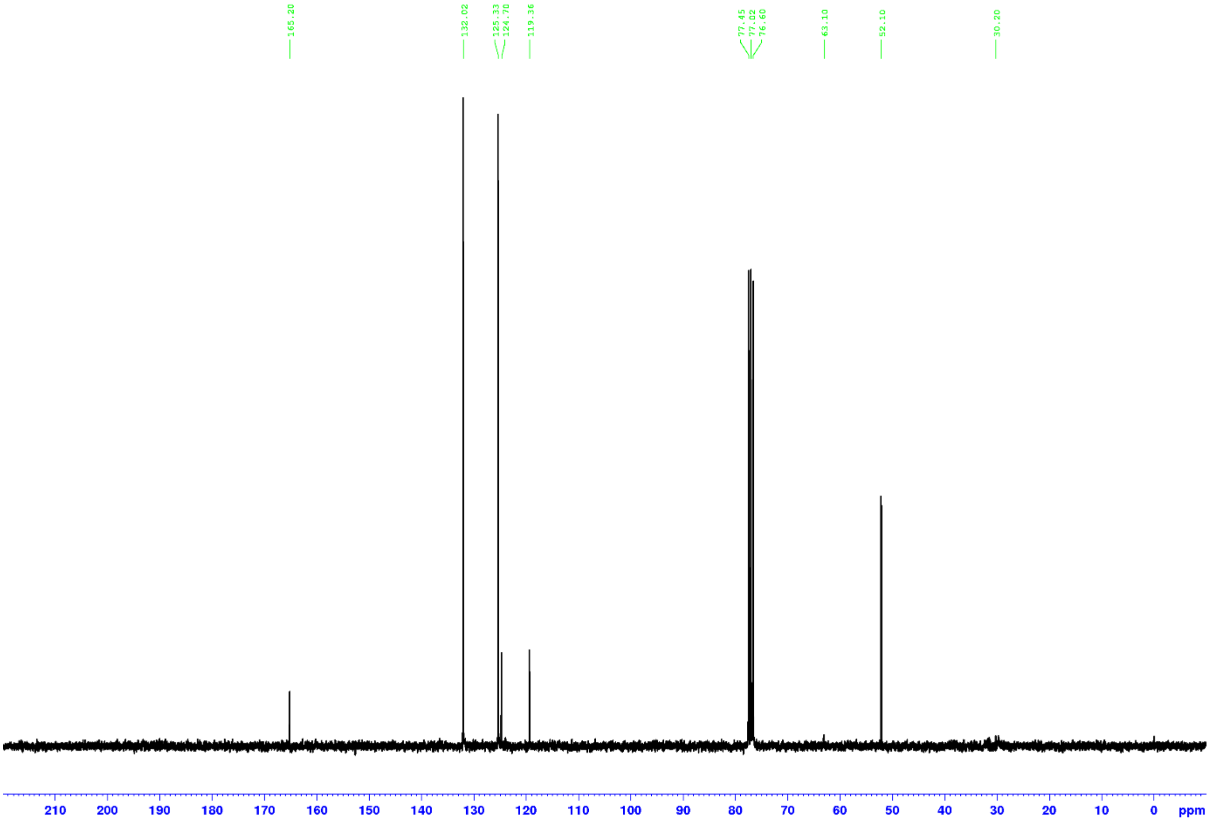

Figure S10. $^{13}C\{^{1}H\}$ NMR ($CDCl_3$,75.5 MHz) spectrum of α-diazo ester **24**.

Diethyl 2-diazomalonate (26)[4]

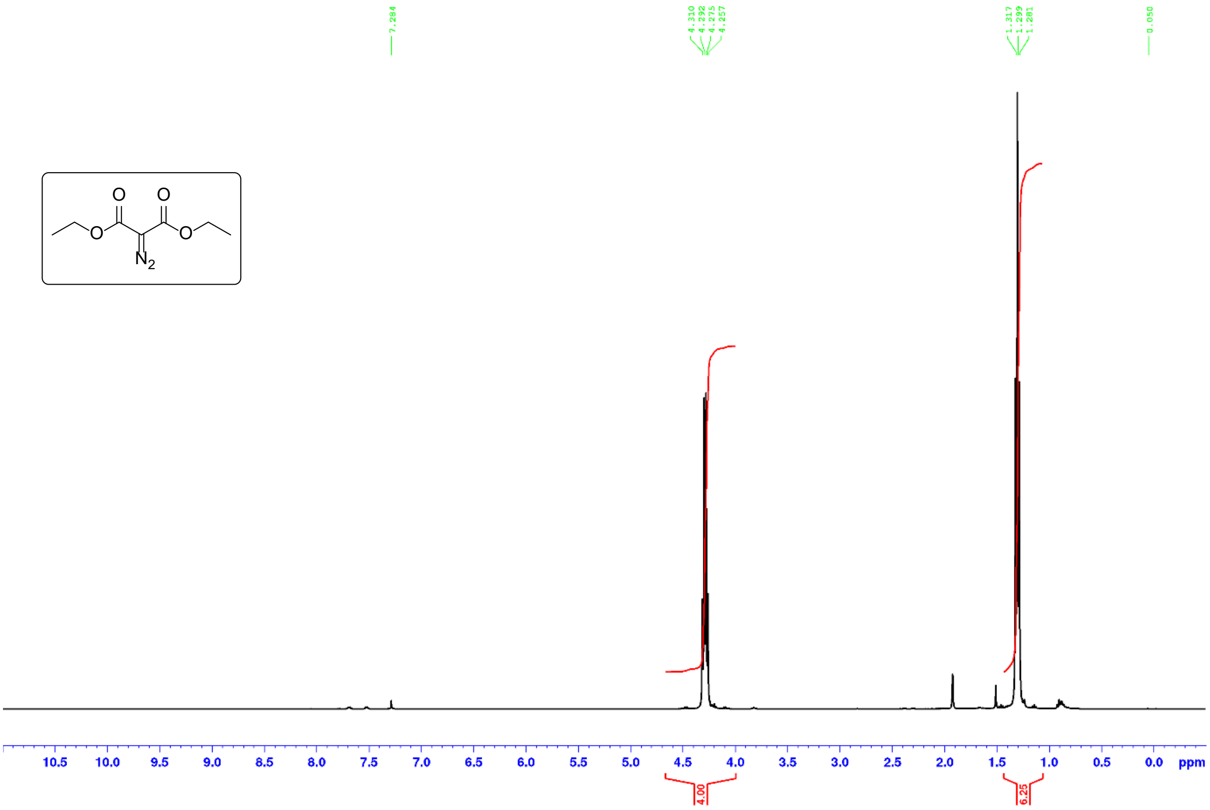

Figure S11. ^{1}H NMR (400 MHz, $CDCl_3$) spectrum of α-diazo ester **26**.

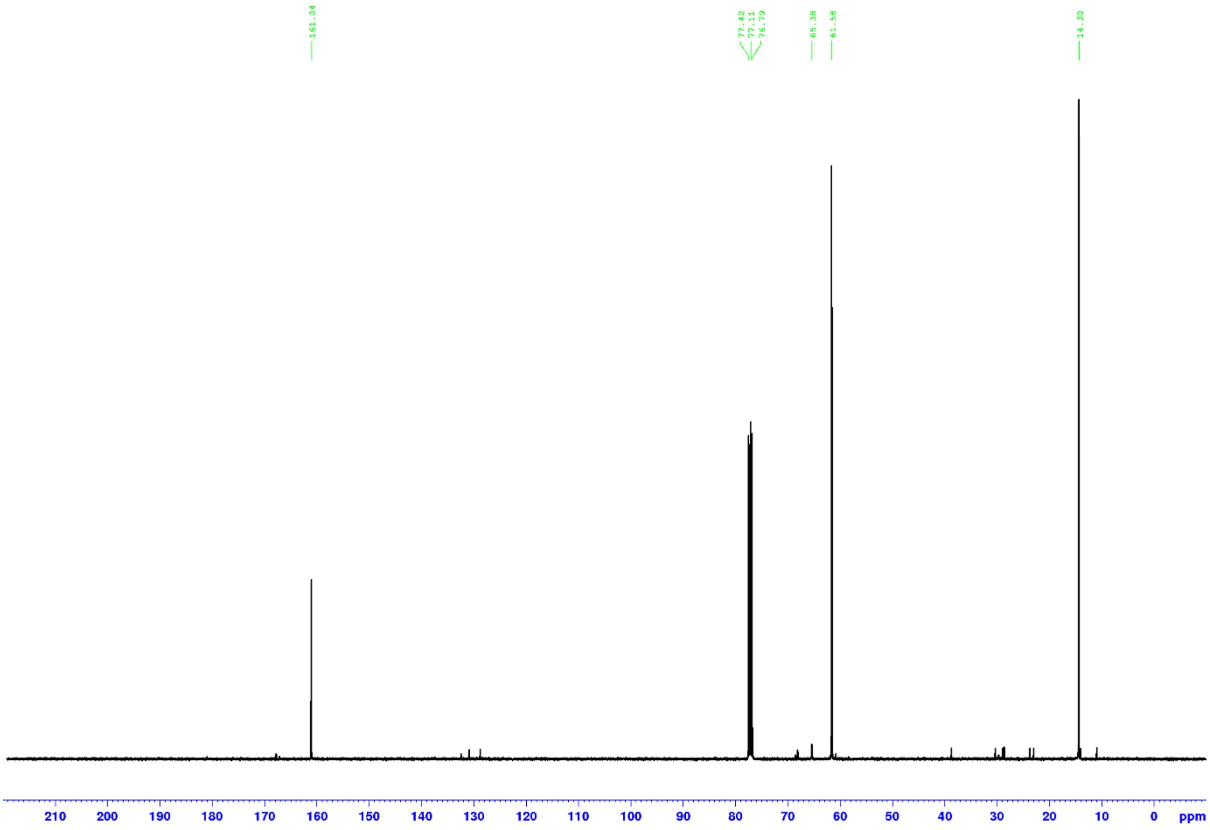

Figure S12. ^{13}C NMR (100.6 MHz, $CDCl_3$) spectrum of α-diazo ester **26**.

Methyl 2-(2-chlorophenyl)-2-hydroxyacetate (10)[5]

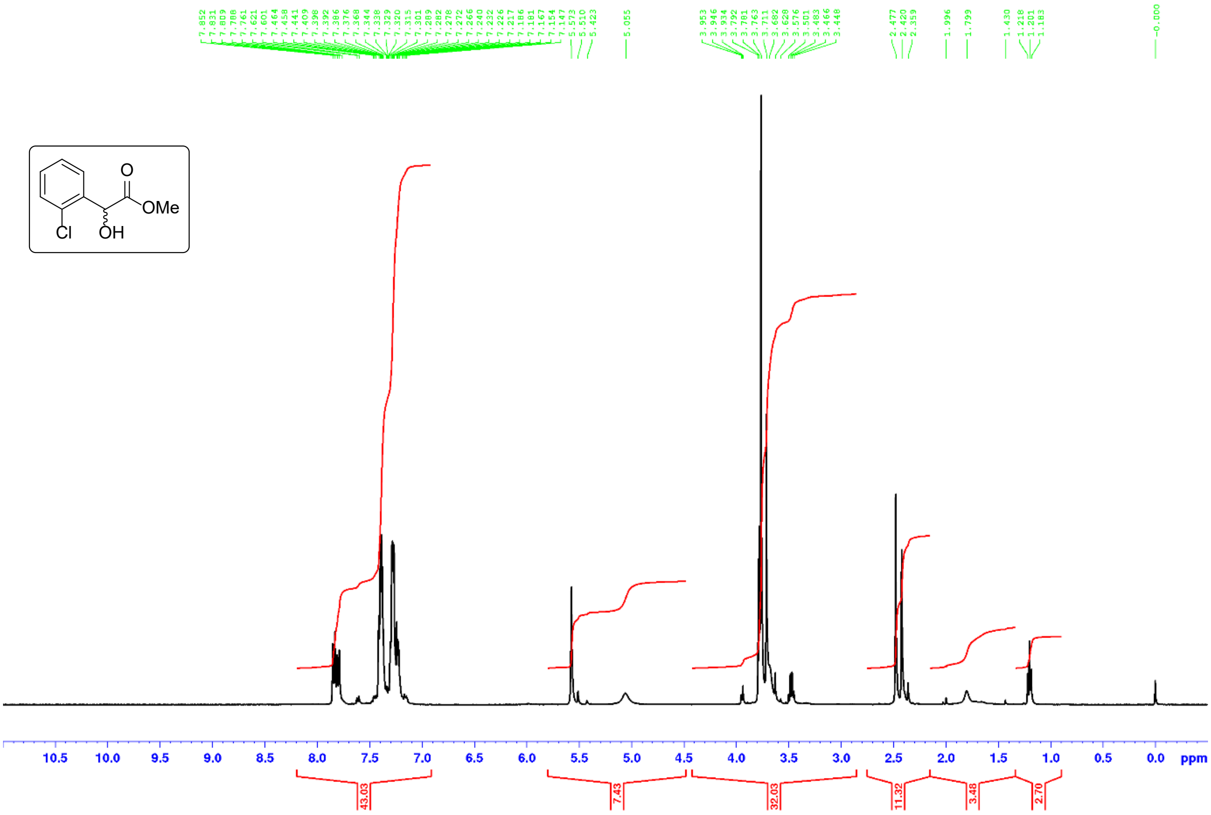

Figure S13. ^{1}H NMR (400 MHz, $CDCl_3$) spectrum of crude α-hydroxy ester **10** (from telescoped process, see Scheme 10).

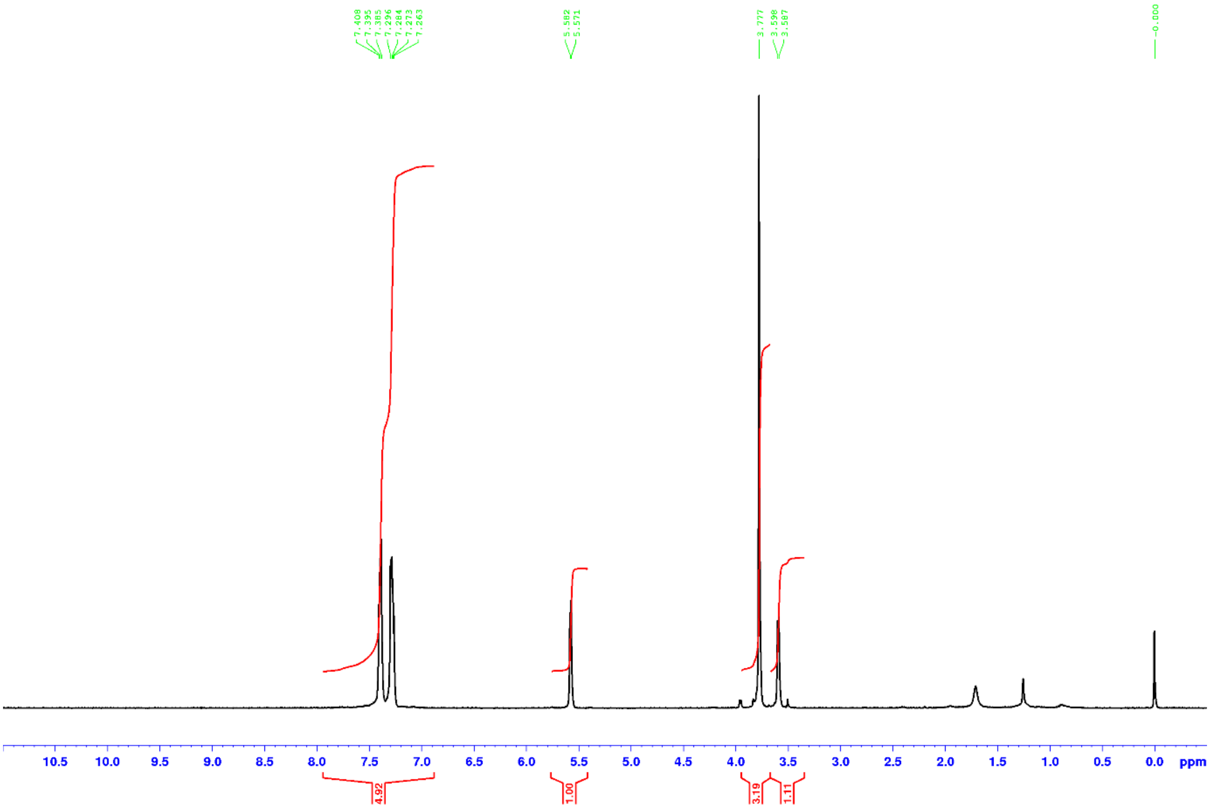

Figure S14. ^{1}H NMR (400 MHz, $CDCl_3$) spectrum of α-hydroxyl ester **10** (from telescoped process, see Scheme 10).

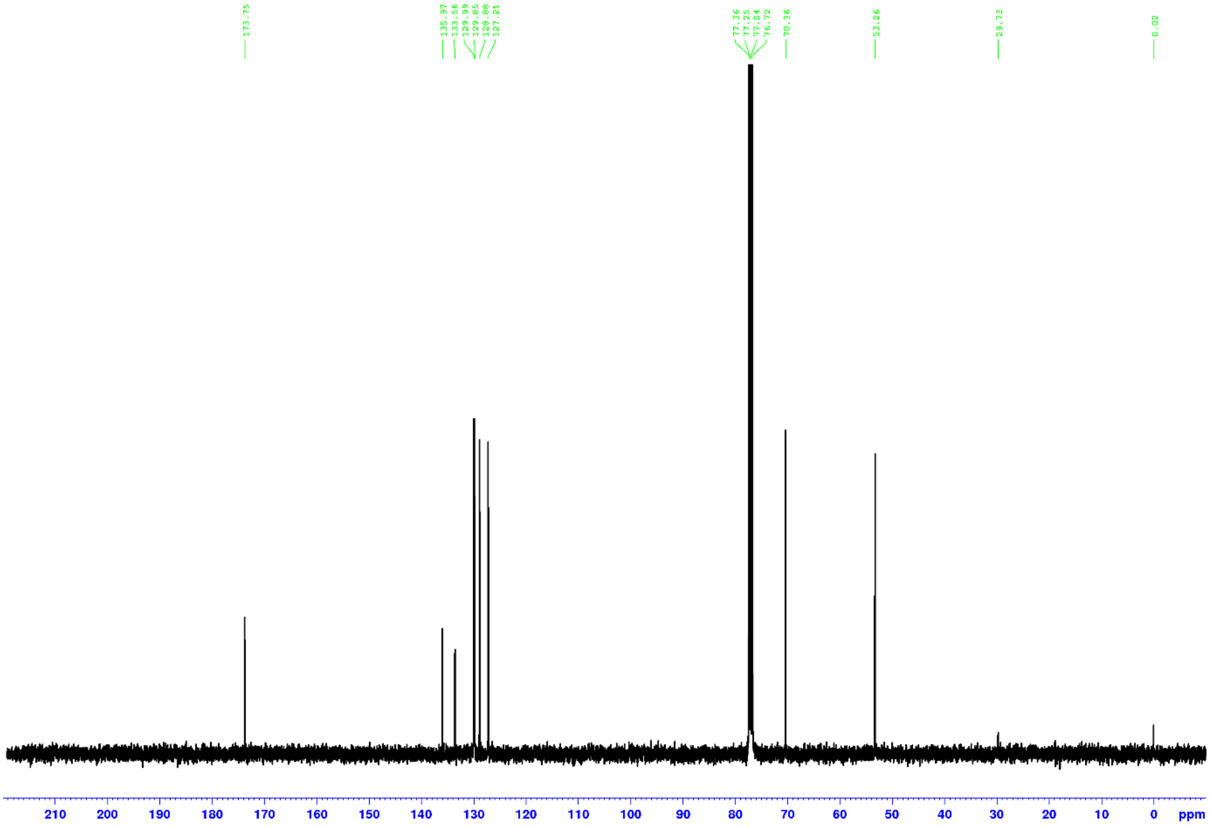

Figure S15. ^{13}C NMR (100.6 MHz, $CDCl_3$) spectrum of α-hydroxyl ester **10** (from telescoped process, see Scheme 10).

.

References

(1) Tayama, E.; Saito, S. Copper-Catalyzed Regiospecific and 1,2-Regioselective Cyclopropanation of (1Z)-1-Amino- and (1Z)-1-Oxy-1,3-butadienyl Derivatives *Synlett*, **2015**, *26*, 1880–1884.

(2) Bartrum, H. E.; Blakemore, D. C.; Moody, C. J.; Hayes, C. J. Rapid Access to α-Alkoxy and α-Amino Acid Derivatives through Safe Continuous-Flow Generation of Diazoesters. *Chem. – Eur. J.*, **2011**, *17*, 9586–9589.

(3) Tayama, E.; Saito, S. Regioselective synthesis of secondary 1,3-dienamides by successive eliminations *Tetrahedron* **2016**, *72*, 599–604.

(4) Kitamura, M.; Tashiro, N.; Okauchi, T. 2-Azido-1,3-dimethylimidazolinium Chloride: An Efficient Diazo Transfer Reagent for 1,3-Dicarbonyl Compounds*Synlett,* **2009**, 2943–2944.

(5) Lloyd-Jones, G. C.; Wall, P. D.; Slaughter, J. L.; Parker, A. J.; Laffan, D. P. Enantioselective homoallyl-cyclopropanation of dibenzylideneacetone by modified allylindium halide reagents—rapid access to enantioenriched 1-styryl-norcarene. *Tetrahedron* **2006**, *62*, 11402–11412.
